# Extracellular vesicles of human transformed skin‐derived precursors containing miR‐221‐3p promote hair growth through DKK2‐mediated Wnt/ *β* ‐catenin signaling

**DOI:** 10.1002/btm2.70130

**Published:** 2026-03-30

**Authors:** Lingyun Zhao, Anqi Li, Shijing Chen, Wei Hua, Ru Dai, Lingyu Pan, Meng Hu, Nan Huang, Runke Zhou, Yuanyuan Han, Lidan Xiong, Li Li

**Affiliations:** ^1^ Department of Dermatology West China Hospital, Sichuan University Chengdu Sichuan China; ^2^ Medical Cosmetic Center, Chengdu Second People's Hospital, Sichuan University Chengdu Sichuan China; ^3^ Department of Dermatology West China Second Hospital, Sichuan University Chengdu Sichuan China; ^4^ Evaluation Center for Cosmetics Safety and Efficacy, West China Hospital, Sichuan University Chengdu Sichuan China; ^5^ Department of Dermatology Second Affiliated Hospital of Zhejiang University School of Medicine, Zhejiang University Hangzhou China; ^6^ State Key Laboratory of Biotherapy, West China Hospital, Sichuan University Chengdu Sichuan China

**Keywords:** DKK2, extracellular vesicles, hair growth, miR‐221‐3p, skin‐derived precursors

## Abstract

Stem cells and their paracrine factors hold promise for alopecia treatment, yet research on human skin‐derived precursors (hSKPs), which are closely related to hair follicles in biological positioning and function, remains limited. We demonstrated that extracellular vesicles of human transformed skin‐derived precursors (htSKP‐EVs), harvested utilizing our directed induction, culture transition and gradient ultracentrifugation technology, exhibited superior efficiency and quality determined by transmission electron microscopy, nanoparticle tracking analysis, and detection of specific markers. Using CCK8, scratch assay, immunofluorescence, H&E staining, immunohistochemistry staining, dermoscope, qRT‐PCR and Western blotting, it was found that htSKP‐EVs significantly promoted the proliferation of hair follicle stem cells (hHFSCs) by effectively modulating the Wnt signaling pathway, thereby enhancing overall hair follicle growth. Notably, miR‐221‐3p, highly expressed in htSKP‐EVs, suppressed DKK2 expression, activated the Wnt pathway in human dermal papilla cells (hDPCs), and induced hair follicles to enter and sustain the anagen phase, based on the aforementioned similar in vivo and in vitro experiments. These findings, validated in hHFSCs, hDPCs and human hair follicles in vitro and in a murine alopecia model in vivo, revealed the potential mechanism of htSKP‐EVs in hair growth and identified a new therapeutic target for alopecia in regenerative medicine.

**Translational Impact Statement**

Extracellular vesicle of human skin‐derived precursors (hSKP‐EVs) offers a promising approach for alopecia treatment. However, the limited quantity of SKPs obtained from human samples and their characteristic slow response as stem cells make culture and passage extremely challenging. We established a directed induction and culture transition technology to obtain human transformed skin‐derived precursors (htSKPs) and a staged gradient ultracentrifugation technology to obtain their EVs (htSKP‐EVs). htSKP‐EVs obtained through this bioengineering approach not only addressed the aforementioned issues but also demonstrated hair growth‐promoting effects supported by multiple lines of evidence from in vivo and in vitro investigations, highlighting its potential for clinical translation.

**Abbreviations**
AGA: androgenetic alopecia
cAMP: cyclic adenosine monophosphate
C‐myc: v‐myc avian myelocytomatosis viral oncogene homolog
DKK2: Dickkopf WNT signaling pathway inhibitor 2
EVs: extracellular vesicles
GWAS: a large‐scale meta‐analysis of genome‐wide association study
H&E: hematoxylin and eosin
hDPCs: human dermal papilla cells
hFBs: human fibroblasts
hHFSCs: human hair follicle stem cells
htSKPs: human transformed skin‐derived precursors
IHC: immunohistochemical
KEGG: Kyoto Encyclopedia of Genes and Genomes
LEF1: lymphoid enhancer binding factor 1
miRNA: microRNA
TCF3: transcription factor 3

## 1 INTRODUCTION

Hair loss, clinically termed alopecia, affects millions of individuals worldwide, carries a substantial psychosocial burden, and is linked to diverse underlying medical conditions.^1^ Current first‐line treatments, including minoxidil and finasteride, have limited clinical efficacy and are frequently accompanied by undesirable side effects.^2^ Researchers continue to investigate the mechanisms governing hair follicle cycling and develop small‐molecule drugs,^3^, ^4^, ^5^ bioproducts,^6^ topical formulations,^7^, ^8^ laser therapy,^9^ and surgical interventions.^10^ Among the emerging strategies under preclinical and clinical investigation, regenerative medicine approaches hold particular promise, as they harness the regenerative potential of stem cells and their secreted factors to promote hair growth and follicle regeneration.

Fibroblasts (FBs), skin‐derived precursors (SKPs), and their secreted extracellular vesicles (EVs) have emerged as pivotal mediators of skin regeneration and hair growth, with significant implications for tissue engineering and therapeutic development. FBs, the predominant cell type in the dermis, are considered by some studies to represent a subset of mesenchymal stem cells (MSCs),^11^ they are valued for their high accessibility and capacity to synthesize extracellular matrix components, generate bioelectric signals, and secrete growth factors, all of which contribute to skin repair and homeostasis.^12^, ^13^ SKPs, isolated from the dermis, exhibit greater differentiation potential and superior neural function recovery capacity compared to other skin‐derived cells, including FBs.^14^ Previous studies have demonstrated that co‐cultured mixtures of SKPs, mesenchymal cells, and epithelial stem cells are capable of forming de novo hair follicles following in vivo injection.^15^, ^16^

However, despite their remarkable differentiation potential, SKPs encounter critical limitations in large‐scale expansion, primarily due to their inherent propensity to form cell aggregates and undergo senescence. In this context, our study introduces an innovative strategy that combines adherent and suspension culture techniques to generate human transformed SKPs (htSKPs), and we further isolate their extracellular vesicles (htSKP‐EVs) via gradient ultracentrifugation. This dual‐step method aims to overcome the drawbacks of traditional cultivation approaches, yielding a more potent and scalable source of therapeutic EVs. Moreover, compared to cellular therapies, EVs exhibit lower immunogenicity and reduced tumorigenic risk in clinical applications.^17^

Our previous and unpublished work has demonstrated that transformed SKPs possess enhanced proliferative capacity and sustained stemness retention compared to conventionally cultured SKPs.^18^ We selected human fibroblast‐derived extracellular vesicles (hFB‐EVs) as the control group based on two key considerations: first, hFB‐EVs have well‐documented roles in skin regeneration and hair growth.^19^, ^20^, ^21^, ^22^, ^23^ This provides a robust benchmark for evaluating the superior efficacy of htSKP‐EVs; second, comparative studies across EVs derived from diverse cellular sources remain extremely scarce in this field. Additionally, hFBs represent an intermediate cell phenotype during htSKP induction, so using hFB‐EVs as a control enables us to more effectively analyze the cellular changes induced by our culture technique, thereby clarifying the underlying mechanisms regulating EV‐mediated hair growth promotion.

In recent years, scientists have increasingly recognized that stem cells exert their regulatory effects on target cells primarily via EVs.^24^ EVs are lipid bilayer vesicles secreted by various cell types, with a diameter of 40–160 nm, and carry abundant biological cargo, including mRNA, miRNA, ncRNA, circRNA, mtDNA, protein ligands, receptors, and transcription factors. MicroRNAs (miRNAs) are a subclass of short non‐coding RNAs that regulate post‐transcriptional gene expression; numerous human protein‐coding genes—closely involved in biological processes such as cell growth, apoptosis, proliferation, inflammation, and immune responses—are regulated by miRNAs.^25^ While several studies have reported correlations between miRNAs and androgenetic alopecia (AGA),^26^, ^27^, ^28^ the underlying molecular mechanisms remain incompletely elucidated. miR‐221‐3p has been detected in various malignant tumors, including breast cancer,^29^ hepatocellular carcinoma,^30^ and prostate cancer,^31^ and can activate the Wnt pathway, promote tumor cell growth and cell cycle progression, and inhibit apoptosis. However, whether miR‐221‐3p modulates the Wnt/*β*‐catenin signaling pathway in hair follicles remains unclear, and its target cells and cognate mRNAs within the hair follicle microenvironment also require further investigation.

To date, the research and application of SKP‐derived EVs for hair growth have not been reported. We conducted a relatively comprehensive investigation into the isolation, characterization, safety, and efficacy of htSKP‐EVs using both in vitro and in vivo experiments. Ultimately, we demonstrated that htSKP‐EVs containing miR‐221‐3p can promote hair growth via the DKK2‐mediated Wnt/β‐catenin signaling pathway, which may provide a novel regenerative medicine strategy and therapeutic target for alopecia.

## 2 MATERIALS AND METHODS

### 2.1 Animal and human ethics

All experiments involving live rodents conformed to appropriate governmental and institutional regulations and were performed according to the guidelines of the Animal Ethics Committee of West China Hospital of Sichuan University (approved animal protocol number: 2017064A and 2021356A) and conducted in accordance with the ARRIVE 2.0 guidelines (https://arriveguidelines.org/arrive-guidelines). The human fibroblasts (hFBs) were obtained from fresh adult discarded foreskin specimens. The human dermal papilla cells (hDPCs), hair follicle stem cells (hHFSCs), and in vitro hair follicles were all derived from the discarded scalp tissue of patients with scalp trauma after surgery (patients signed informed consent); approximately 20–40 hair follicles were collected from each tissue in the occipital area using follicular unit extraction techniques, and the ethical approval number was 2019 Review (143), which met the ethical requirements of West China Hospital of Sichuan University.

### 2.2 Isolation, culture and identification of htSKPs

Foreskins were obtained from healthy young males after circumcision, disinfected with iodophor and 75% ethanol, and rinsed three times with phosphate‐buffered saline (PBS) containing 1% PS (penicillin/streptomycin double antibiotic, Hyclone, USA). The subcutaneous fat layer was removed, retaining the dermis and epidermis. The skin was cut into 5 × 5 mm pieces and digested overnight with 0.25% trypsin (Hyclone, USA) in a 4°C refrigerator. The epidermis was rinsed twice with PBS to separate and wash it off, and any unseparated epidermis was manually removed with tweezers. The dermal tissue was cut into 2 × 3 mm pieces, and 1 mg/mL type I collagenase (Hyclone, USA) was added for digestion at 37°C in an incubator for 2 h until the tissue was almost completely digested into a chylous state. Digestion was terminated by adding 5 volumes of high‐glucose DMEM medium (Hyclone, USA). The cells were mechanically dissociated by repeated pipetting with a 1 mL pipette tip. The above suspension was filtered through a 40 μm cell strainer, and the filtrate was centrifuged at 1500 rpm for 5 min. After discarding the supernatant, the cells were resuspended in complete medium, counted with a cell counter, seeded in a culture dish at a density of 4 × 10^4^ cells/mL, and incubated at 37°C with 5% CO_2_ and 95% air.

After digestion, adherent fibroblasts were centrifuged, and the cell pellet was resuspended in tSKP medium: high‐glucose DMEM medium (Hyclone, USA)/F12 nutrient supplement (Gibco, USA) = 3:1, supplemented with 40 ng/mL bFGF, 20 ng/mL EGF (Millipore, USA), 2% B27 (Gibco, USA), and 1% PS. The cells were seeded in a culture flask coated with Poly‐HEMA (Sigma, USA) at a density of 2–4 × 10^4^ cells/mL. Every 3–4 days, 1–2 mL of DMEM/F12 (3:1) medium supplemented with all growth factors was added to maintain the htSKPs medium composition (40 ng/mL bFGF, 20 ng/mL EGF, and 2% B27). Continuous culture was performed for 1–2 weeks, and after centrifugation, htSKPs and the medium supernatant were collected for subsequent use.

The morphology of htSKPs was observed daily under an optical microscope. After htSKPs formed cell spheres, the number of clonal spheres was counted using a microscope (Olympus, Japan). Fields with more than 3 SKPs per 100× field of view were selected for photography, 5 fields were recorded, and the maximum diameter of clonal spheres in each field was measured using ImageJ software.

To identify the multi‐directional differentiation potential of htSKPs, adipogenic, osteogenic, and myogenic differentiation experiments were performed.^32^, ^33^

### 2.3 Extraction and identification of htSKP‐EVs

When htSKPs were cultured in 25 cm^2^ flasks for 7–10 days, reaching approximately 90% cell density with the maximum number of uniformly sized cell spheres, the cells were centrifuged at 2000 g for 10 min at 4°C. The supernatant was discarded, and the cell pellet was retained, resuspended in high‐glucose DMEM medium without fetal bovine serum, and cultured for an additional 48 h. Subsequently, differential centrifugation was performed at 4°C: sequential centrifugation at 300*g* and 2000*g* for 10 min each, followed by centrifugation at 10,000*g* for 30 min, collecting the supernatant after each step. The supernatant was transferred to ultracentrifuge tubes for floor‐standing ultracentrifuges and centrifuged at 100,000*g* for 70 min at 4°C. The pellet was collected, resuspended in PBS, filtered through a 0.22 μm filter, and transferred back to ultracentrifuge tubes for a second centrifugation at 100,000*g* for 70 min at 4°C. The supernatant was discarded, and the pellet was resuspended in 200 μL PBS using a micropipette to obtain htSKP‐EVs.

The size and concentration of htSKP‐EVs were determined using a ZetaView nanoparticle tracking analyzer (NTA). The extracted htSKP‐EVs were diluted in ultrapure water at a ratio of 1:1000 to 1:2000 (adjusted according to the actual EV concentration). A 1 mL syringe was used to draw the diluted htSKP‐EVs and inject them into the sample inlet of the NTA instrument. Sample results were analyzed using ZetaView software (version 8.05.11), and the experiment was repeated three times. Western blot (WB) was used to identify EV‐specific markers (CD9, CD63, CD81) and the negative marker calnexin (Abcam, USA, 1:1000).

### 2.4 In vitro functional assays of htSKP‐EVs

The CCK‐8 assay (Dojindo, Japan) was used to measure hHFSC proliferation. Briefly, CCK‐8 solution was added to cells in a 96‐well plate, incubated for 1–4 h, and the absorbance at 450 nm was measured. The test concentrations of htSKP‐EVs were divided into low concentration (1 × 10^9^ particles/mL, 2 μL) and high concentration (1 × 10^10^ particles/mL, 2 μL). An equal volume of PBS (the solvent for htSKP‐EVs) was used as the blank control, and minoxidil (a mainstream anti‐hair loss drug) served as the positive control.

Primary hDPCs and hHFSCs were isolated and cultured by microdissection combined with enzymatic digestion. hDPCs were cultured in CM‐H249 medium (Pricella) and hHFSCs in CM‐H237 medium (Pricella) until forming a confluent monolayer. A scratch was made on the cell layer, detached cell debris was removed, and culture medium was added. Cell migration in the scratch area was observed and recorded at different time points, and the migration capacity was evaluated by measuring the change in scratch width using image analysis software.

WB was used to detect the expression of Wnt pathway‐related proteins in hHFSCs (all antibodies were purchased from Abcam, USA, 1:1000), following the procedures of cell lysis, protein quantification, SDS‐PAGE electrophoresis, membrane transfer, blocking, primary and secondary antibody incubation, and protein band detection as described above.

Human hair follicles in the anagen phase were selected for subsequent experiments, characterized by a bulbous, deeply stained, matrix‐rich dermal papilla, with an overall inverted wine glass shape. These ex vivo human hair follicles can be continuously cultured for up to 30 days.

After intervention with htSKP‐EVs in ex vivo human hair follicle culture, changes in hair follicle growth length and dermal papilla diameter were observed using an optical microscope.

### 2.5 In vivo functional assays of htSKP‐EVs

Establishment of hair removal‐induced hair cycle alopecia model: Female C57BL/6 mice aged 6–7 weeks (SiPeiFu, China) were anesthetized via intraperitoneal injection of 5% chloral hydrate (0.1 mL/10 g). After anesthesia, the dorsal hair was removed with a razor, and an area of 4 × 2.5 cm, approximately 1 mm thick depilatory cream was applied to the shaved region. The hair removal cream (Veet, France) was left for 5 min before being wiped off with water to remove both the cream and hair. C57 mice of 6–7 weeks with hair follicles in the telogen phase have skin that appears pinkish‐white, indicating successful model establishment.

Randomization: mice that met the inclusion criteria for the depilation‐induced hair loss model were stratified by baseline hair‐cycle score and body weight to balance potential confounders, then randomly allocated to equal‐sized groups using a computer‐generated random‐number list prepared by an investigator who was blinded to the treatment codes.

Sample sizes for all animal experiments were pre‐validated using G*Power 3.1 software, ensuring an effect size of ~0.8 or higher to meet the accepted standard for adequate statistical power to detect true group differences.

Post‐injection of fluorescently labeled (CM‐Dil, 25 μg/mL, Maokang Biotechnology) ht‐SKPs‐EVs: Three mice from the hair cycle model group were injected subcutaneously with prepared exosomes at a concentration of 1 × 10^10^ particles/ml in PBS, using a 9‐point method (arranged around the center of the mouse's back, with as even spacing as possible), with 0.1 mL per mouse. The mice and their biodistribution were then imaged using the IVIS Spectrum (Caliper Life Sciences) at 0, 24, 48, 72 h post‐injection, with all fluorescence intensities analyzed using software.

Twenty successfully modeled mice were evenly divided into five groups, with four mice per group. The groups are: Blank control with PBS, htSKP‐EVs group, hFB‐EVs group, and Minoxidil group. After successful modeling, mice were anesthetized with 5% chloral hydrate (0.1 mL/10 g) via intraperitoneal injection. Subsequently, they were injected subcutaneously with either PBS, htSKP‐EVs (1 × 10^10^ particles/mL), hFB‐EVs (1 × 10^10^ particles/mL), or SKPs (1 × 10^6^ cells/mL), each at a volume of 0.1 mL. The injections were administered every 2 days. The positive control group applied 2% Minoxidil solution (Aladdin, China) topically twice daily in the morning and evening. The animal experiment was repeated three times following the aforementioned method.

### 2.6 Protein mass spectrometry

Proteomic analysis of htSKP‐EVs and hFB‐EVs was performed using an Orbitrap Astral mass spectrometer (Thermo Fisher Scientific, USA). Briefly, proteins were extracted from EV samples, reduced, alkylated, and digested into peptides with trypsin. The resulting peptides were separated by liquid chromatography (LC) and analyzed by tandem mass spectrometry (LC–MS/MS). Protein identification and quantification were conducted using Proteome Discoverer 2.2 (PD2.2, Thermo Fisher Scientific) with database searches against the NCBInr, UniProt, and BioGRID databases. Student's *t*‐test was used for statistical analysis of protein quantification data between groups. Differentially expressed proteins (DEPs) were defined as those with *p* <0.05 and |log₂FC| >2. Functional annotation of proteins and DEPs was performed using InterProScan software for Gene Ontology (GO) and IPR (InterPro) terms, incorporating databases including Pfam, PRINTS, ProDom, SMART, ProSite, and PANTHER. Additionally, Clusters of Orthologous Groups (COG) analysis was conducted for identified proteins, and DEPs were subjected to volcano plot visualization and Kyoto Encyclopedia of Genes and Genomes (KEGG) pathway enrichment analysis.

### 2.7 miRNA sequencing

miRNA sequencing of htSKP‐EVs and hFB‐EVs was carried out using an Illumina HiSeq 2500 platform (Illumina, USA). Total RNA, including small RNAs, was isolated from EV samples. Library construction was performed following standard protocols, and high‐throughput sequencing was conducted to identify and quantify miRNAs. After quality filtering, length distribution statistics were generated for total and unique clean reads of both groups. Pearson correlation analysis was used to evaluate differential miRNA expression, with screening thresholds set at |log₂(FoldChange)| >2 and *q*‐value <0.05. GOseq was employed to calculate the enrichment probability of differential miRNAs in GO terms, and the KEGG pathway database was used to analyze molecular pathways enriched by differential miRNAs.

### 2.8 Target gene prediction and validation of miR‐221‐3p

Target genes of miR‐221‐3p were predicted using four bioinformatics databases: miRBase, miRDB, miRWalk, and TargetScan. A Venn diagram was constructed to visualize the overlapping target genes across the four databases, and 191 intersecting genes were obtained. Functional enrichment analysis of these target genes was performed using Metascape and DAVID, incorporating resources such as KEGG Pathway, GO Biological Processes, Reactome Pathway Database, Canonical Pathways, and CORUM. Based on enrichment results and literature reports, DKK2 was identified as a potential target gene of miR‐221‐3p. Base‐pairing between miR‐221‐3p and the DKK2 mRNA transcript was analyzed using TargetScan, and dual‐luciferase reporter assays were conducted to validate the targeting relationship.

hDPCs were transfected with miR‐221‐3p‐mimic or miR‐221‐3p‐inhibitor (Hanbio Biotechnology, China). Quantitative real‐time PCR (qRT‐PCR) was performed to verify the transfection efficiency of miR‐221‐3p, following the protocol described below.

### 2.9 In vitro functional assays of miR‐221‐3p

hDPCs were treated with different concentrations of miR‐221‐3p‐mimic, alongside a negative control (mimic‐NC) and positive control (minoxidil). Cell proliferation was measured at 24, 48, and 72 h using a CCK‐8 assay kit. Based on transfection efficiency results, the 60 nM miR‐221‐3p‐mimic group and minoxidil group were selected for scratch wound healing assays, with observations recorded at 0, 24, and 48 h post‐scratch to evaluate cell migration. After 72 h of treatment, WB was used to detect the expression of DKK2 (a negative regulator of the Wnt/β‐catenin pathway; Bio‐Techne, USA) and downstream pathway‐related proteins, including *β*‐catenin, LEF1 (Abcam, USA, 1:1000), c‐Myc, and CyclinD1 (SAB, USA, 1:1000).

### 2.10 In vivo functional assays of miR‐221‐3p

Thirty female C57BL/6 mice (6–7 weeks old, SiPeiFu, China) with successful hair cycle modeling were randomly divided into five groups (*n* = 6 per group) using the same randomization method as above. Each mouse received subcutaneous injections at 8 sites on the dorsal skin (0.5 cm apart, 0.05 mL per site) on days 0, 7, 14, and 21. The positive control group received topical 2% minoxidil solution twice daily (morning and evening).

### 2.11 Gross observation and dermoscopy

Every 7 days, mice were anesthetized by intraperitoneal injection of 1% sodium pentobarbital (0.1 mL/10 g body weight). Gross photographs of the dorsal skin were taken, and dermoscopic examination (CBS ELECTRONIC, China) was performed using both polarized and non‐polarized light modes to observe hair follicle growth and skin color changes.

### 2.12 WB analysis

Cells or tissues were washed with PBS and lysed in RIPA lysis buffer supplemented with a protease inhibitor cocktail (1:100, Beyotime, China). The supernatant was collected as total protein extract and stored at −80°C. Protein concentration was determined using a BCA protein assay kit (Beyotime, China) with a standard curve. Protein samples were mixed with loading buffer, denatured by boiling, and separated by SDS‐PAGE electrophoresis. Electrophoresis buffer was prepared by dissolving 3.03 g Tris, 14.4 g glycine, and 1 g SDS in distilled water to a final volume of 1 L. Samples and a molecular weight marker were loaded onto the gel, which was run at 80 V to align the protein bands, then at 120 V for 40–50 min until marker bands were clearly visible.

Proteins were transferred from the gel to a PVDF membrane (Millipore, USA) using transfer buffer (3.03 g Tris, 14.4 g glycine, 200 mL methanol, and distilled water to 1 L). To detect proteins with distinct molecular weights while preserving sample integrity, membranes were cut according to the molecular weight marker prior to blocking. For proteins with similar molecular weights, membranes were incubated with the first primary antibody, detected, and then stripped using a stripping buffer (Beyotime, China) following the manufacturer's instructions to remove bound antibodies, enabling re‐probing with a second primary antibody.

Membranes were blocked to prevent non‐specific binding, then incubated with primary antibodies overnight at 4°C, followed by secondary antibodies at room temperature. Protein bands were visualized using an ECL chemiluminescent substrate (Beyotime, China) and imaged with an Invitrogen iBright imaging system (Thermo Fisher Scientific, USA). Band optical density was quantified to evaluate protein expression levels.

### 2.13 Hematoxylin and eosin (H&E) staining

Every 7 days, one mouse per group was euthanized, and a 1 cm × 1 cm dorsal skin sample was collected. Tissues were fixed in 4% paraformaldehyde for 24 h, then dehydrated with gradient ethanol, cleared with xylene, and embedded in paraffin to prepare paraffin blocks. Serial 5‐μm‐thick sections were cut along the longitudinal axis of the skin and hair follicles, mounted on glass slides, and baked at 60°C for 30 min. After dewaxing with xylene and rehydration with gradient ethanol, sections were stained with hematoxylin for 2 min and 0.5% eosin for 2 min. Stained sections were observed and photographed under an inverted microscope to assess hair cycle changes.

### 2.14 Immunohistochemical (IHC) staining

Five‐micrometer‐thick sections were dewaxed and rehydrated as described in the H&E staining protocol. Antigen retrieval was performed by incubating sections in citrate buffer. Non‐specific binding sites were blocked with 5% bovine serum albumin (BSA) for 30 min at room temperature. Sections were then incubated with primary antibodies overnight at 4°C, followed by incubation with secondary antibodies and chromogenic substrate. Images were captured to evaluate the effect of htSKP‐EVs on the expression of hair growth‐related proteins.

### 2.15 Quantitative real‐time PCR (qRT‐PCR)

Total RNA was isolated from hHFSCs using a standard kit. One microgram of total RNA was reverse‐transcribed into cDNA using a reverse transcription kit (Vazyme, China). qRT‐PCR was performed using the SYBR Premix Ex Taq II kit (TAKARA, Japan) on a LightCycler 480 System (Roche, Switzerland), following the manufacturer's protocol and a previously reported method.^34^ Primers for Wnt pathway‐related genes were designed using Primer Premier 5.0 software (Tsingke Biotech, China), and sequences are listed in Table S1.

### 2.16 Dual‐luciferase reporter assay

Wild‐type (h‐DKK2‐3′UTR‐wt) and mutant (h‐DKK2‐3′UTR‐mu) fragments of the DKK2 3′UTR containing the predicted miR‐221‐3p binding site were synthesized (Hanbio, China) and inserted into the pSI‐Check2 vector using HB Infusion™ Master Mix (Hanbio, China). 293T cells (Hanbio, China) were co‐transfected with miR‐221‐3p‐mimic and either h‐DKK2‐3′UTR‐wt or h‐DKK2‐3′UTR‐mu. Control groups included cells co‐transfected with mimic‐NC and h‐DKK2‐3′UTR‐wt, or mimic‐NC and h‐DKK2‐3′UTR‐mu. After 48 h of incubation, luciferase activity was measured using the Promega Dual‐Luciferase Reporter Assay System according to the manufacturer's instructions.

### 2.17 Microarray data deposition

miRNA sequencing data have been submitted to the Sequence Read Archive under accession number PRJNA1194988. Mass spectrometry proteomics data have been deposited to the ProteomeXchange Consortium via the iProX partner repository with the dataset identifier PXD058677.

### 2.18 Statistical analysis

Statistical computations were carried out using SPSS 16.0 (IBM, USA) and Origin 9.0 (OriginLab, USA) software. All experiments were performed with at least three independent biological replicates and three technical replicates per sample. Prior to parametric testing, all datasets were subjected to Shapiro–Wilk normality assessment and Levene's test for variance homogeneity to satisfy the underlying assumptions of parametric analyses. For comparisons between two independent cohorts, two‐tailed unpaired Student's *t*‐test was employed when normality and variance homogeneity were confirmed; in cases where these assumptions were violated, the non‐parametric Mann–Whitney U test was adopted as an alternative. For multi‐group comparisons, one‐way analysis of variance (ANOVA) was performed after verifying normality and variance homogeneity, with Tukey's HSD test utilized for post‐hoc pairwise comparisons to mitigate Type I errors associated with multiple testing. All data are expressed as mean ± standard deviation (SD), and the results were considered significant at *p <0.05, ** *p* <0.01, ***p <0.001.

## 3 RESULTS

### 3.1 HtSKPs and htSKP‐EVs exhibit the biological characteristics of native SKPs

**FIGURE 1:**
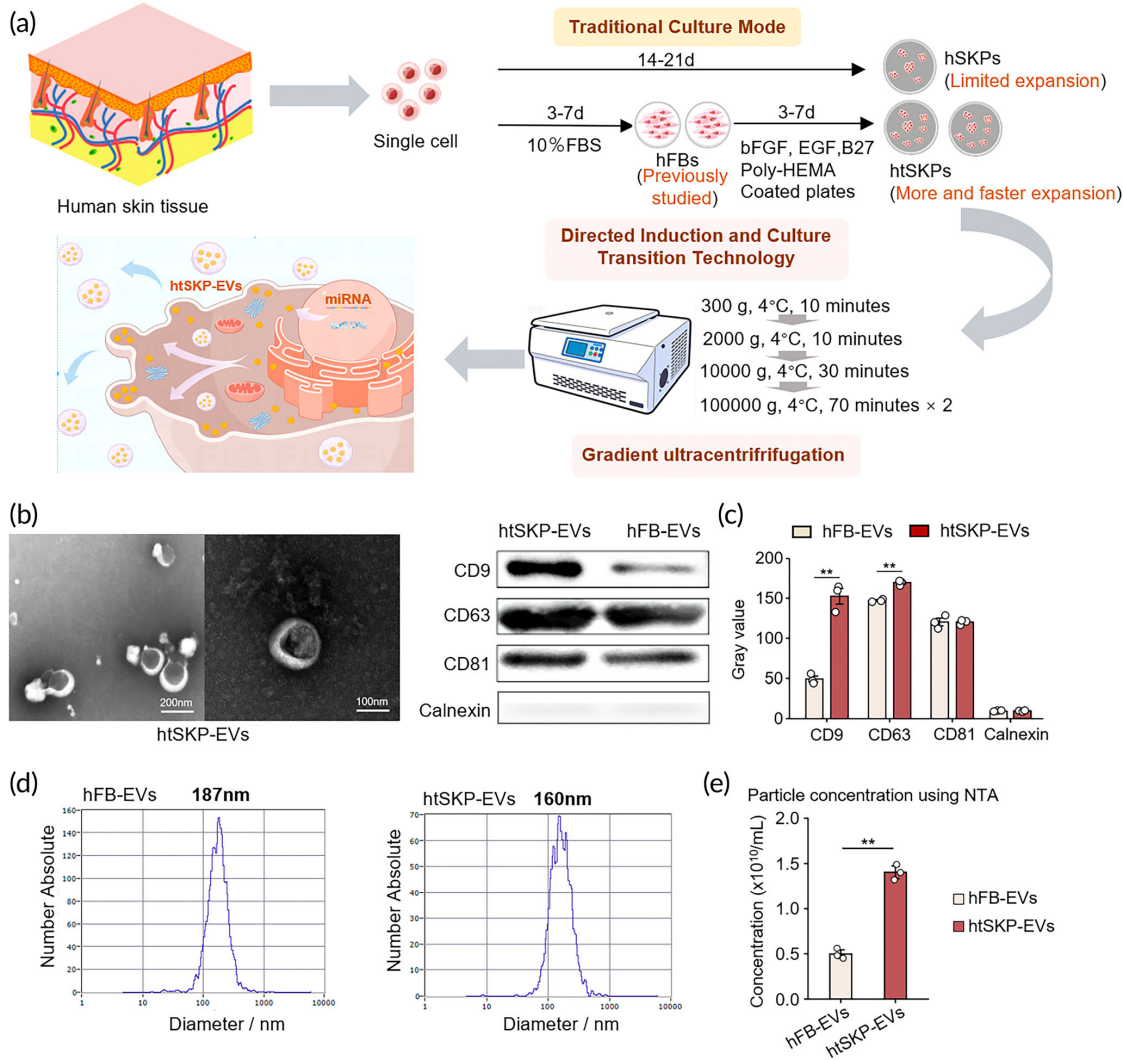
Obtainment and identification of htSKPs and htSKP‐EVs. (a) This flowchart describes the process of obtaining htSKPs and htSKP‐EVs. (b) Representative TEM images of htSKP‐EVs showing intact cup‐shaped morphology and bilayer membrane (left, 200 nm; right, 100 nm). (c) Expression of EV markers. Western blot showed that htSKP‐EVs had high expression levels of CD9/CD63 markers. And there was no significant difference in the expression of CD81 in hFB‐EVs (*n* = 3). (d) Particle size distribution of hFB‐EVs and htSKP‐EVs. The particle size distribution of the two types of EV is consistent, without too small and too large particle size. (e) Particle concentration of hFB‐EVs and htSKP‐EVs using NTA. (*n* = 3) Data were presented as the mean ± SD. Statistical analyses were performed using unpaired two‐tailed Student's *t* tests, with significant differences observed (**p <0.01). EVs, extracellular vesicles; htSKPs, human transformed skin‐derived precursors; hFBs, human fibroblasts; NTA, nanoparticle tracking analysis; TEM, transmission electron microscopy.

The htSKPs exhibited the biological characteristics of native SKPs, with the advantages of faster culture and higher cell yield. They transformed from adherent growth (hFBs) to suspended clonal spheres (htSKPs) within approximately 3–7 days (a total of 6–14 days), which was more rapid than the formation of clonal spheres (SKPs) via traditional direct suspension culture (Figure 1a, S1). Moreover, htSKPs possessed stem cell properties, including the potential for osteogenic, adipogenic, and myofibroblastic differentiation (Figure S2). Under transmission electron microscopy, htSKP‐EVs presented a typical saucer‐like morphology (Figure 1b). The expression of EVs biomarkers (CD9, CD63, and CD81) in htSKP‐EVs was comparable to or higher than that in hFB‐EVs, while the negative control marker calnexin was barely expressed (Figure 1c). In addition, the diameter distributions of htSKP‐EVs and hFB‐EVs were similar (Figure 1d). The concentration of purified htSKP‐EVs was approximately 1.4 × 10^10^ particles/mL, which was about threefold higher than that of hFB‐EVs isolated under the same conditions and in the same period (Figure 1e).

### 3.2 HtSKP‐EVs promote the proliferation of hHFSCs, hDPCs and in vitro hair follicles by activating the Wnt/*β*‐catenin pathway

**FIGURE 2:**
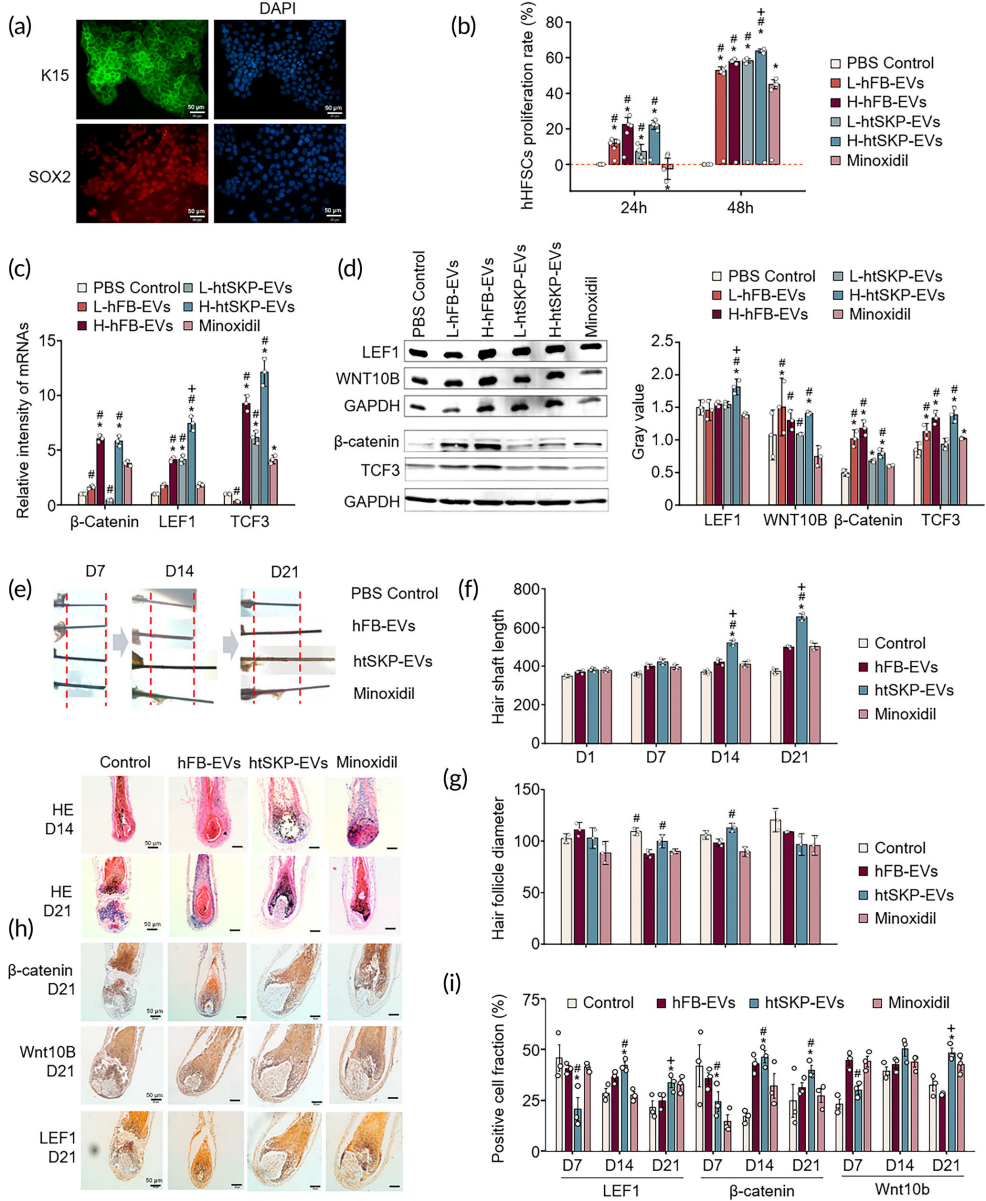
htSKP‐EVs can promote the proliferation of hHFSCs and in vitro hair follicles by activating the Wnt/*β*‐catenin pathway. (a) The image shows the cell proliferation rate of different treatment groups. (b) Cellular immunofluorescence identification of human HFSCs specific markers. Above: K15 (green fluorescence) nuclear staining (blue fluorescence); Below: SOX2 (red fluorescence) nuclear DAPI staining (blue fluorescence) (scale = 100 nm). (c) Gene expression of HFSCs in Wnt signal pathway with different treatments (*n* = 3). (d) Protein expression of hHFSCs in Wnt signal pathway with different treatments. The upper image represents the protein band optical density values. The lower image shows the Western blot protein bands (*n* = 3). (e) Morphological change of in vitro hair follicle with different treatments (*n* = 3). (f) and (g) Comparison of hair shaft length and Hair follicle diameter with time in different treatment groups (*n* = 3). (h) Hematoxylin and eosin staining and immunohistochemical images of hair follicles in vitro (scale = 50 μm). (i) Immunohistochemical quantized results of LEF1, *β*‐Catenin, TCF3 (*n* = 3). L and H represent low and high concentrations, 10^9^ and 10^10^ particles/mL, respectively; (mean ± SD; one‐way ANOVA; * indicates *p* <0.05 compared with the PBS control group; # indicates *p* <0.05 compared with the minoxidil group. + indicates *p* <0.05 compared with the hFB‐EVs group). DAPI, 4′,6‐diamidino‐2′‐phenylindole; EVs, extracellular vesicles; htSKPs, human transformed skin‐derived precursors; hFB, human fibroblasts; hHFSCs; human hair follicle stem cells.

Isolated hDPCs and hHFSCs were identified by specific immunofluorescence markers (hDPCs: *β*‐catenin, ALP, Figure S3; hHFSCs: K15, SOX2, Figure 2a). After 48 h of treatment with htSKP‐EVs, hHFSCs displayed a small, cuboidal or polygonal morphology with smooth surfaces and large nuclei containing diffuse chromatin, accompanied by a significant upregulation of KRT14 expression (*p* <0.01); these features collectively indicated high proliferative and metabolic activity (Figures S4, S5). The proliferative effect of htSKP‐EVs on hHFSCs was concentration‐dependent, with the high‐concentration group (H‐htSKP‐EVs, 1 × 10^10^ particles/mL, 2 μL) showing the highest proliferation rate at 48 h among all tested groups (Figure 2b). htSKP‐EVs moderately enhanced the proliferation of human DPCs, whereas the scratch wound healing assay revealed no obvious effect on their migratory ability (Figure S6).

Analysis of relative mRNA expression levels demonstrated that htSKP‐EVs exerted a more significant regulatory effect on TCF3 and LEF1 than hFB‐EVs and minoxidil. Both htSKP‐EVs and hFB‐EVs robustly upregulated *β*‐catenin expression, with a stronger effect at higher concentrations compared to minoxidil (*p* <0.05) (Figure 2c). WB results showed that high concentrations of htSKP‐EVs markedly increased the expression of Wnt pathway‐related proteins; notably, htSKP‐EVs specifically enhanced LEF1 expression, while hFB‐EVs had a more pronounced effect on *β*‐catenin (*p*<0.05). No significant difference in TCF3 and WNT10B expression was observed between the htSKP‐EVs and hFB‐EVs groups, but both groups exhibited higher expression levels than the PBS control and minoxidil groups (*p* <0.05) (Figure 2d).

In vitro hair follicle culture experiments showed that htSKP‐EVs promoted the transition of hair follicles to the anagen phase earlier than hFB‐EVs and minoxidil, and the anagen phase was sustained until day 21 (Figure 2e,f). The most obvious change in hair follicle diameter occurred at 14–21 days in vitro, which was consistent with the observed changes in hair shaft growth. Notably, the hair follicle diameter in the htSKP‐EVs group was larger than that in the minoxidil group at day 14 (Figure 2g). H&E staining revealed that from day 14 onwards, the htSKP‐EVs group exhibited enlarged dermal papillae and abundant hair matrix, suggesting an earlier transition into the anagen phase (Figure 2h). IHC staining showed that the expression of WNT10B and LEF1 in the htSKP‐EVs group was significantly higher than that in the hFB‐EVs and minoxidil groups (*p* <0.05). No significant difference in *β*‐catenin expression was found between the htSKP‐EVs and hFB‐EVs groups, but both groups had stronger expression than the minoxidil group (*p* <0.05) (Figure 2h,i; all original images in Figures S7–S10).

### 3.3 HtSKP‐EVs promote the entry of telogen hair follicles into anagen in mice by regulating the Wnt/*β*‐catenin pathway

**FIGURE 3:**
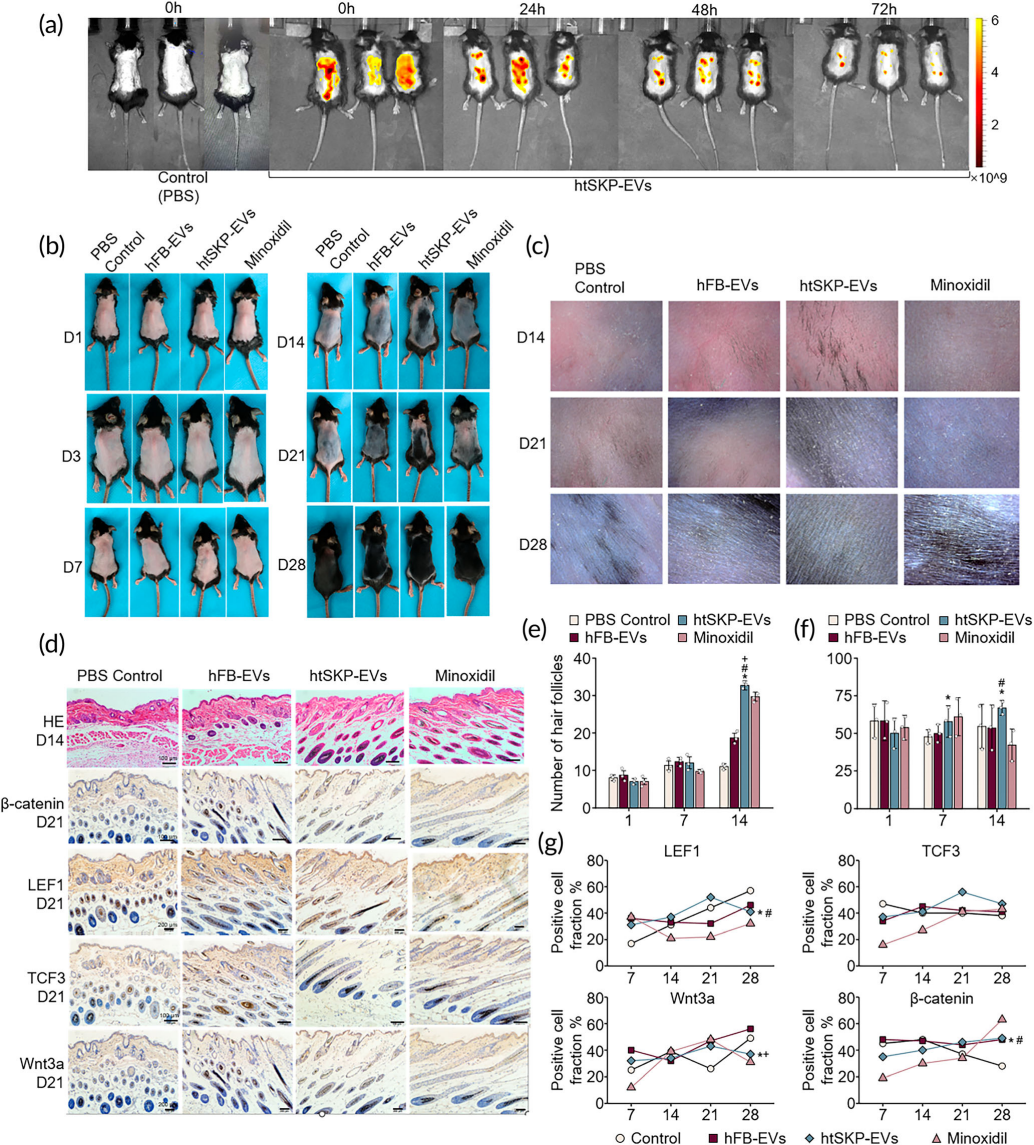
htSKP‐EVs promote hair growth in mice by regulating the Wnt/*β*‐catenin pathway. (a) In vivo imaging confirmed that htSKP‐EVs were almost completely absorbed and metabolized after 72 h (*n* = 3). (b) General images of the mice alopecia model in each group (*n* = 4). (c) Dermoscopic observations confirmed this result in day 14, 21, 28 (*n* = 4). (d) Hematoxylin and eosin staining and immunohistochemical images of the mice alopecia model in each group (scale = 100 and 200 μm). (e) and (f) Comparison of the diameter and number of hair follicles with time in different treatment groups (*n* = 3). (g) Immunohistochemical quantized staining results of *β*‐Catenin, TCF3, WNT3A, and LEF1 (*n* = 3) (mean ± SD; one‐way ANOVA;* indicates *p* <0.05 compared with the PBS control group; # indicates *p* <0.05 compared with the Minoxidil group; + indicates *p* <0.01 compared with the hFB‐EVs group); EVs: extracellular vesicles; htSKPs: human transformed skin‐derived precursors.

A murine hair cycle model was successfully established by shaving combined with depilatory cream application. In vivo imaging confirmed that htSKP‐EVs (1 × 10^10^ particles/mL in PBS, 0.1 mL per mouse) were almost completely absorbed and metabolized at 72 h post‐injection (Figure 3a). A strong fluorescent signal of htSKP‐EVs was first detected in the liver at 24 h post‐injection, with a weaker signal in the lungs and no detectable fluorescence in other organs. Additionally, the hepatic fluorescent signal remained at a high intensity at 48 h post‐injection. These observations indicated that the liver is the primary organ for htSKP‐EVs metabolism, which is consistent with the classic retention and clearance mechanisms of EVs (Figure S11). Based on dose–response experiments (Figure S12), a concentration of 1 × 10^10^ particles/mL was selected for subsequent in vivo studies.

From day 14 onwards, the dorsal skin color of mice in all groups turned gray, indicating the initiation of the anagen phase, with the most obvious change observed in the htSKP‐EVs group (Figure 3b). Dermoscopic analysis further confirmed this finding, showing a significantly higher emergence of new hair shafts in the htSKP‐EVs group at day 14 (Figure 3c). H&E staining revealed that with the extension of intervention time, hair follicles became deeper and their numbers gradually increased (Figure 3d). At day 14, the htSKP‐EVs group still had the largest hair follicle diameter and the highest hair follicle number, suggesting a delayed entry into the catagen phase (Figure 3e,f). No pathological changes were observed in organ sections of mice in the htSKP‐EVs group (Figure S13). IHC staining confirmed the upregulated expression of *β*‐catenin, TCF3, WNT3A, and LEF1 in the htSKP‐EVs‐treated group (*p* <0.05), indicating that the Wnt signaling pathway is involved in the regulation of hair cycle by htSKP‐EVs (Figure 3d,g; all original images in Figures S14–S18).

### 3.4 Protein mass spectrometry of htSKP‐EVs reveals biomarker enrichment associated with the Wnt pathway

**FIGURE 4:**
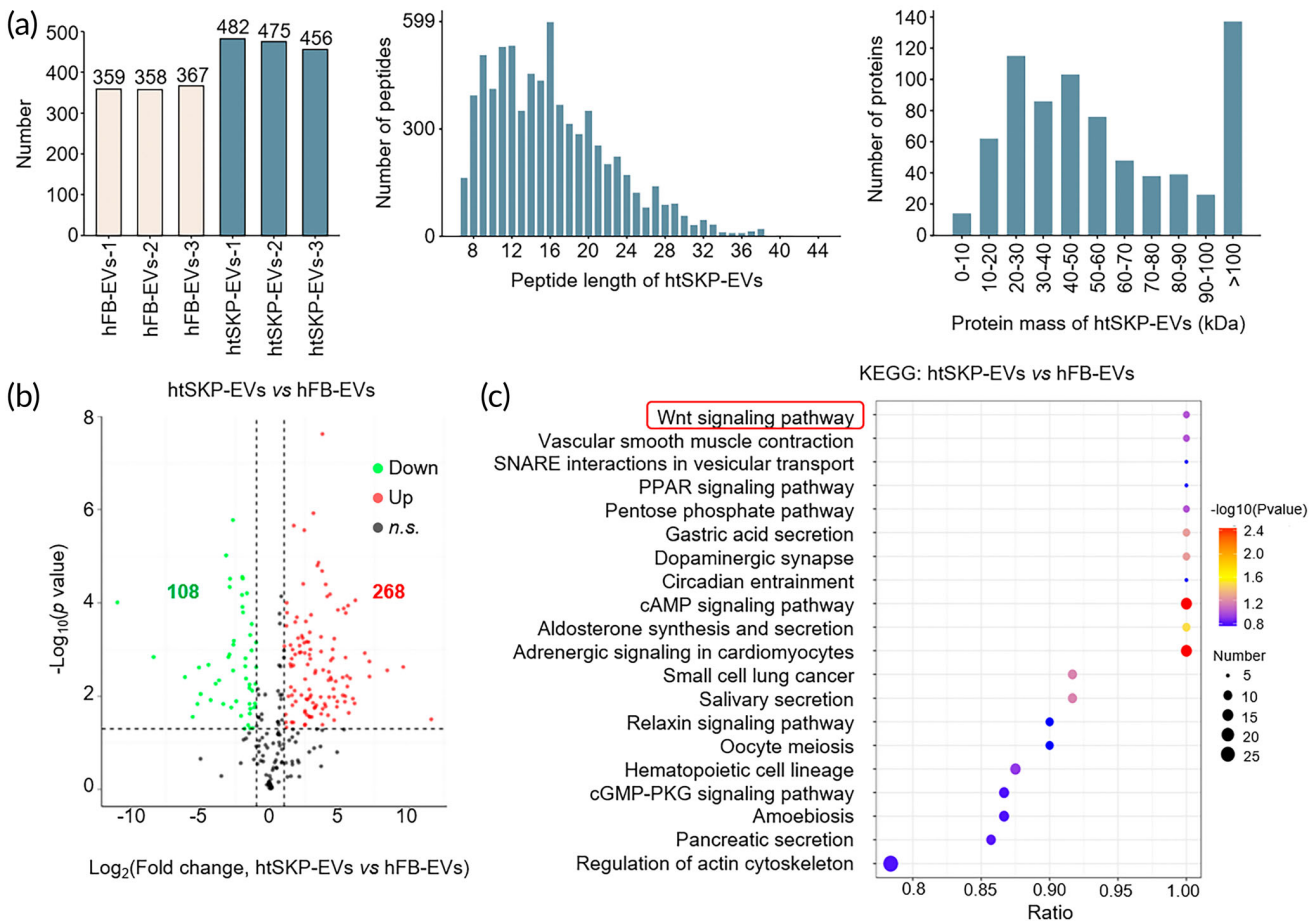
Protein mass spectrometry for htSKP‐EVs revealed biomarker enrichment associated with the Wnt pathway. (a) Protein mass spectrometry showed that the protein number of htSKP‐EVs was higher than that of hFB‐EVs, and the peptide length of htSKP‐EVs was mainly between 7 and 28, protein mass was mainly 20–30 KDa and more than 100 kda. (b) The volcano map made by differential protein analysis between the two showed that there were 477 differentially expressed proteins, of which 268 were more than twice up‐expressed and 108 were more than twice down‐expressed. (c) Analysis of Kyoto Encyclopedia of Genes and Genomes (KEGG) showed that Wnt signaling pathway (Blue frame mark) pathway were highly enriched signaling pathways. (*n* = 3); EVs, extracellular vesicles; htSKPs, human transformed skin‐derived precursors; hFBs, human fibroblasts; n.s., not significant.

Protein mass spectrometry analysis showed that the number of proteins identified in htSKP‐EVs was higher than that in hFB‐EVs. The peptide length of htSKP‐EVs was predominantly 7–28 amino acids, with molecular weights mainly distributed at 20–30 kDa and above 100 kDa (Figure 4a). GO and COG annotation analysis indicated that the main functions of htSKP‐EVs‐derived proteins were protein binding and post‐translational modification (Figures S19, S20). Volcano plot analysis of differential proteins between htSKP‐EVs and hFB‐EVs identified 477 DEPs, including 268 upregulated and 209 downregulated proteins (Figure 4b). Subsequent KEGG enrichment analysis revealed that these DEPs were significantly enriched in the Wnt signaling pathway, cAMP signaling pathway, and adrenaline signaling pathway (Figure 4c).

### 3.5 MiRNA sequencing of htSKP‐EVs identifies miR‐221‐3p as the most differentially expressed miRNA and its target gene DKK2

Length distribution analysis showed that the majority of RNA fragments in both htSKP‐EVs and hFB‐EVs fell within the canonical miRNA range (18–26 nt) (Figure S21), confirming the successful capture of mature miRNAs. After adapter trimming and quality filtering, 28.1% of the reads from htSKP‐EVs and 16.6% from hFB‐EVs were classified as valid small RNAs, while mRNA‐derived fragments accounted for less than 4% in both groups (Table S2).

**FIGURE 5:**
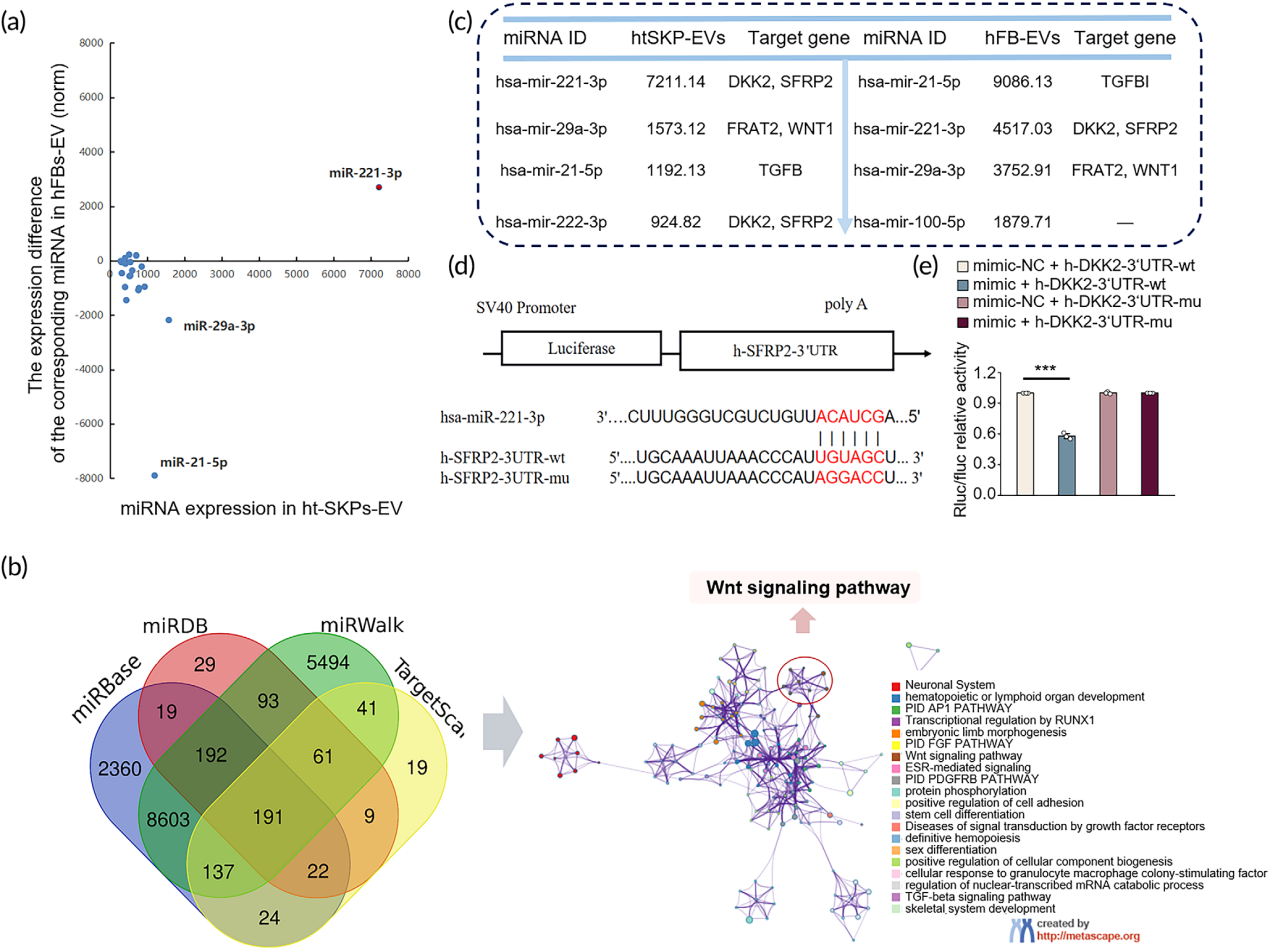
miRNA sequencing for htSKP‐EVs identified miRNA‐221‐3p with the highest differential expression and its target gene DKK2. (a) Sequencing results for miRNA indicate that miR‐221‐3p is not only the most highly expressed in htSKP‐EVs but also shows the greatest upregulation compared to hFB‐EVs. (b) Predicted target genes for miR‐221‐3p include 22,749 in miRBase, 615 in miRDB, 14,812 in miRWalk, and 504 in TargetScan, with an intersection of 191 genes across the four databases. Analysis with DAVID software reveals an enrichment area marked by a red box, indicating the Wnt/*β*‐catenin signaling pathway. (c) Combined with the enriched pathway results and previous literature reports, the predicted target genes of top 5 miRNAs are shown here. (d) Base‐pairing analysis shows that miR‐221‐3p can pair with six bases at one site of the DKK2 mRNA transcript. E. The interaction between miR‐221‐3p and the 3′UTR of h‐DKK2. Compared to the mimic‐NC group, the mimic group significantly downregulated the expression of luciferase driven by h‐DKK2‐3′UTR‐wt (*n* = 3), indicating a binding interaction between the two in this experiment. After mutation, compared to the mimic‐NC group, the mimic group failed to downregulate the expression of luciferase driven by h‐DKK2‐3′UTR‐mu, confirming the accuracy of the mutation sites (mean ± SD; one‐way ANOVA; *** indicates *p* <0.001 compared with mimic‐NC + h‐DKK2‐3′UTR‐wt). EVs, extracellular vesicles; h‐DKK2‐3′UTR‐wt, wild‐type DKK2; h‐DKK2‐3′UTR‐mu, DKK2 with mutated binding sites; htSKPs, human transformed skin‐derived precursors; hFBs, human fibroblasts; NC, negative control.

miRNA sequencing results indicated that miR‐221‐3p was not only the most highly expressed miRNA in htSKP‐EVs but also the most significantly upregulated miRNA compared to hFB‐EVs (Figure 5a). Target gene prediction for miR‐221‐3p identified 22,749 genes in miRBase, 615 in miRDB, 14,812 in miRWalk and 504 in TargetScan, with 191 overlapping genes across the four databases. Subsequent functional enrichment analysis using DAVID software revealed significant enrichment in the Wnt/*β*‐catenin signaling pathway (marked by red box, Figure 5b). Combined with the pathway enrichment results and previous literature reports, the predicted target genes of the top five differentially expressed miRNAs are shown in Figure 5c, with DKK2 identified as the most potential target gene. Base‐pairing analysis demonstrated that miR‐221‐3p can form six base pairs with one site in the DKK2 mRNA transcript (Figure 5d). Dual‐luciferase reporter assays further confirmed the direct targeting relationship between miR‐221‐3p and DKK2, and the predicted binding site was validated (Figure 5e).

### 3.6 In vivo and in vitro experiments confirm that miR‐221‐3p promotes hair growth by downregulating DKK2 and activating the Wnt/*β*‐catenin pathway in hDPCs

**FIGURE 6:**
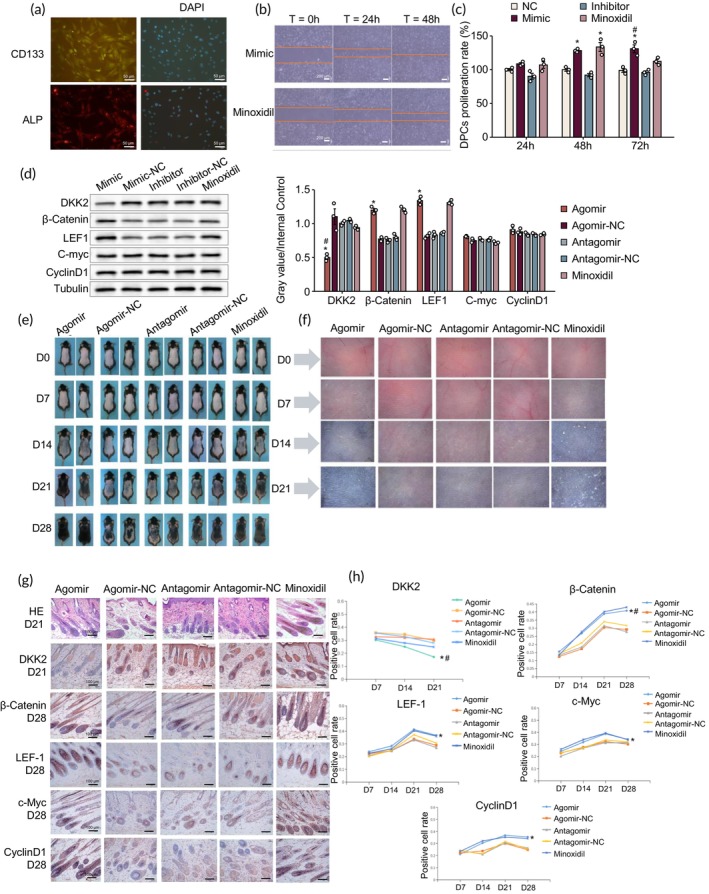
In vivo and in vitro experiments confirmed that miR‐221‐3p promotes hair growth by down‐regulating DKK2 activation of the Wnt/*β*‐catenin pathway in DPCs. (a) The extracted DPCs were identified by specific immunofluorescence markers (CD133 and ALP). (b) Comparison of DPC scratch‐assay results between miR‐221‐3p mimic and minoxidil treatments (scale = 200 μm). (c) The image showed the cell proliferation rate of different treatment groups (*n* = 3). (d) Western blot showed that the expression of DKK2 in the miR‐221‐3p‐mimic group was significantly downregulated, with statistically significant differences compared to the other groups; the expression of *β*‐catenin, LEF1 in the miR‐221‐3p‐mimic group was significantly higher than that in the miR‐221‐3p‐inhibitor group, Minoxidil group, and blank control group (*n* = 3). (e) and (f) In vivo imaging and dermatoscopic observations were similar to previous results, with the miR‐221‐3p‐mimic group mice showing graying of the skin and hair shaft growth on the back, which was noticeable on day 14, and the effect was close to that of the minoxidil group (*n* = 6). (g) Hematoxylin and eosin staining results indicated that by day 21, the Agomir group and the Minoxidil group exhibited better hair follicle quantity and quality compared to the other groups. (g) and (h) Immunohistochemical results indicate that DKK2 expression is downregulated over time in various treatment groups, with the Agomir group showing the most significant decrease, significantly lower than the other groups. The expression levels of *β*‐catenin, LEF1, c‐Myc, and CyclinD1 in the Agomir group and Minoxidil group are higher than those in the blank control group, with the most pronounced difference observed on day 21. (scale = 100 μm; *n* = 3; mean ± SD; one‐way ANOVA; * indicates *p* <0.01 compared with the blank or negative control (NC) group; # indicates *p* <0.05 compared with the positive control group: Minoxidil).

The transfection efficiency of miR‐221‐3p‐mimic in hDPCs was observed to be maximal at a concentration of 60 nM (Figure S22). The isolated hDPCs were identified by specific immunofluorescence markers, CD133 and ALP (Figure 6a). The scratch assay results indicated that the cell gap in the mimic group was essentially closed after 48 h, suggesting that miR‐221‐3p‐mimic significantly promotes the migratory ability of hDPCs (Figure 6b). Both miR‐221‐3p‐mimic and minoxidil could promote the proliferation of hDPCs, with a statistically significant difference observed at 72 h (Figure 6c). After hDPCs were treated separately for 72 h, the expression of DKK2 in the miR‐221‐3p‐mimic group was found to be significantly downregulated compared to the other groups (*p* <0.05); the expression of *β*‐catenin and LEF1 in the miR‐221‐3p‐mimic group was significantly higher than that in the miR‐221‐3p‐inhibitor group, minoxidil group, and PBS control group (*p* <0.05) (Figure 6d).

In vivo gross imaging and dermatoscopic observations were consistent with previous results, with mice in the miR‐221‐3p‐mimic group showing dorsal skin graying and hair shaft growth that was noticeable at day 14. The observed effects were comparable to those in the minoxidil‐treated group (Figure 6e,f).

H&E staining indicated that by day 21, the agomir group and the minoxidil group exhibited superior hair follicle quantity and quality compared to the other groups (Figures 6g and S23). IHC staining showed that DKK2 expression was downregulated over time in all treatment groups (*p* <0.05). Notably, the agomir group exhibited the most significant decrease relative to the other groups. Furthermore, the expression levels of *β*‐catenin, LEF1, c‐myc, and CyclinD1 in the agomir group and minoxidil group were higher than those in the PBS control group, with the most pronounced difference observed at day 21 (*p* <0.05) (Figure 6g,h, all original images in Figures S24–S28). For safety evaluation, mice were euthanized at day 28 after agomir intervention, and multiple organs were stained with H&E. No tumor tissue or pathological changes were found under the microscope (Figure S29).

## 4 DISCUSSION

**FIGURE 7:**
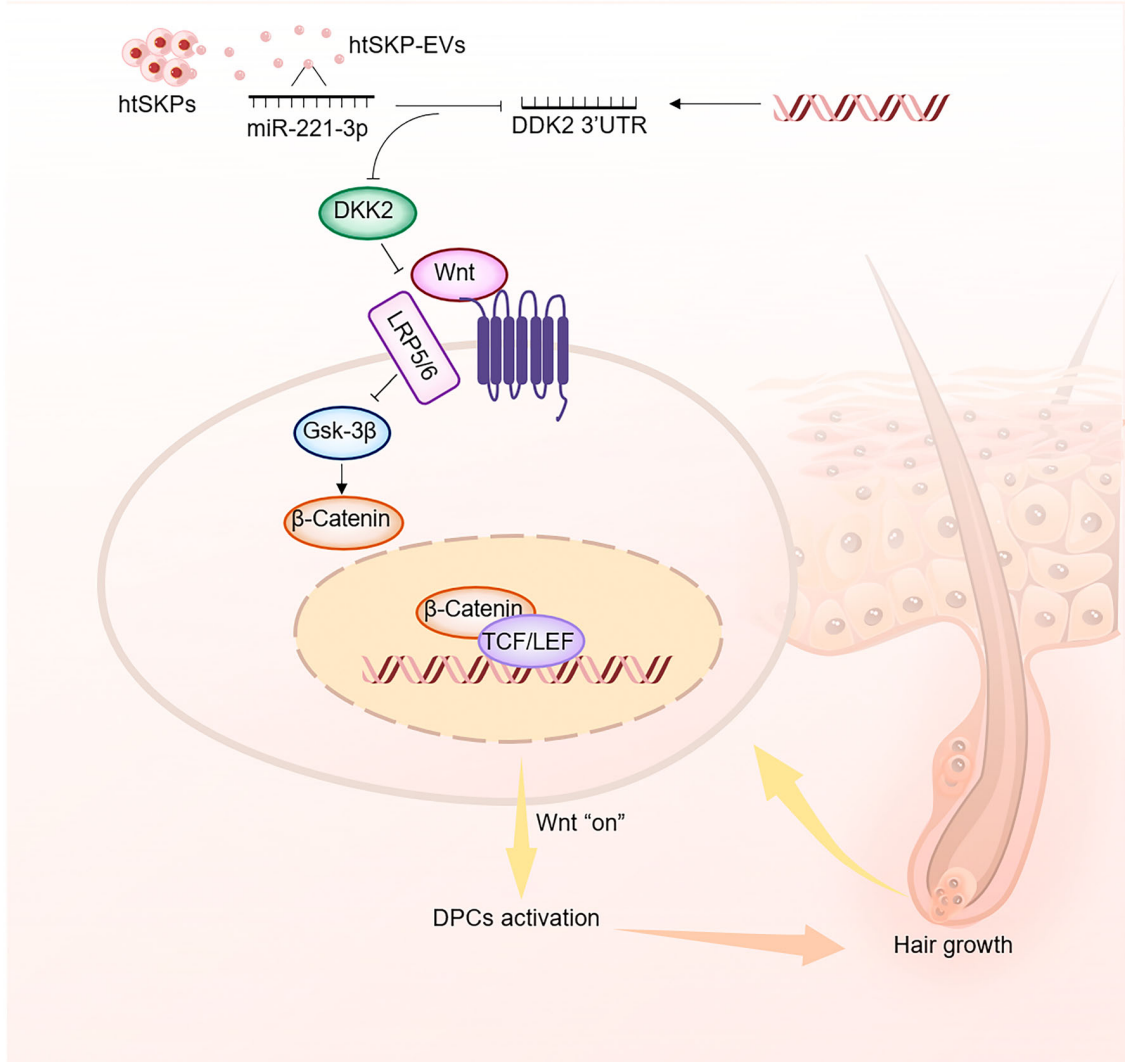
An overview of the mechanisms. The binding of Wnt ligand to its receptor frizzled and co‐receptors LRP5 and 6 (5/6) stabilizes cytosolic *β*‐catenin by suppressing axin function. The stabilized *β*‐catenin translocates into the nucleus to stimulate transcriptional activity by activating the transcription factors TCF1, 3, and 4 and LEF1. DKK2 is a high‐affinity ligand for LRP6 and inhibits Wnt signaling by preventing Fz‐LRP6 complex formation induced by Wnt. miR‐221‐3p from htSKP‐EVs downregulates DKK2 by binding to DDK2 3′UTR, thereby activating the Wnt signaling pathway and positively influencing the hair follicle growth cycle. htSKPs, human transformed skin‐derived precursors; htSKP‐EVs, extracellular vesicles of human transformed skin‐derived precursors; LRP, low density lipoprotein receptor‐related proteins.

Given the limited effectiveness of conventional alopecia treatments, our investigation centered on developing a new biological product, htSKP‐EVs. Initially, we utilized directed induction and culture transition techniques in conjunction with gradient ultracentrifugation to overcome the production challenges of htSKP‐EVs. Our study revealed that htSKP‐EVs promote the proliferation of hHFSCs by modulating the Wnt/*β*‐catenin signaling pathway, thus stimulating hair growth both in vitro and in vivo. The proteins and miRNAs present in htSKP‐EVs facilitate hair follicle development and phase transition by regulating various signaling pathways, including the Wnt pathway. Particularly, miR‐221‐3p, identified through sequencing, was observed to downregulate DKK2 expression, activate the Wnt pathway, and boost cell proliferation and migration upon transfection into hDPCs using miRNA transfection vectors. In a mouse model of alopecia, miR‐221‐3p induced hair follicles to enter and maintain the growth phase compared to the negative control group. An outline of the primary mechanisms is depicted in Figure 7. Collectively, these results endorse htSKP‐EVs as a promising candidate for alopecia treatment.

Human skin serves as a valuable source of various stem cells, positioning it as a promising candidate for research and transplantation. While substantial numbers of rodent SKPs have been successfully cultured, the cultivation of human primary SKPs remains challenging. The current methodology for generating human SKPs (hSKPs) is labor‐intensive and time‐consuming, yielding only a few thousand hSKPs.^35^ Additionally, the suspension spheres often adhere to the plastic surfaces, and premature adherence contributes to a diminished yield of hSKPs.^36^, ^37^, ^38^ To address these issues, we optimized the procedures by treating the flasks with poly‐HEMA, a biomaterial suitable for use as a nonadherent coating.^39^ Currently, conventional methods for isolating EVs, including gradient ultracentrifugation, density gradient centrifugation, and size exclusion chromatography, face challenges related to low yield and contamination with impurities. Building upon the classical gradient ultracentrifugation technique, we significantly enhanced the yield and stability of human SKP‐derived EVs (htSKP‐EVs) and reduced the extraction time for htSKP‐EVs.

Wnt proteins are a family of lipid‐modified secreted glycoproteins that regulate various cellular functions by binding to specific receptors on the cell membrane. This interaction facilitates the translocation of cytoplasmic *β*‐catenin into the nucleus, thereby activating signal transduction pathways in target cells.^40^ Wnt proteins play a crucial role in regulating morphological development during embryogenesis and also participate in the regeneration of various adult tissues, including skin and hair follicle tissue.^41^, ^42^, ^43^ LEF1 is a key factor that promotes the nuclear entry of *β‐*catenin and acts as an effector molecule responsible for the biological functions of β‐catenin.^44^, ^45^, ^46^, ^47^ Our study confirmed through qRT‐PCR and WB that high concentrations of htSKP‐EVs can upregulate LEF1 expression. This finding indicates that htSKP‐EVs influence the Wnt/*β*‐catenin pathway by upregulating LEF1 to regulate the proliferation and growth of hHFSCs. Among the various Wnt proteins, WNT10B uniquely induces the differentiation of epidermal stem cells into hair matrix cells and stimulates the growth of hair shafts in vitro.^47^, ^48^ The significant upregulation of WNT10B expression by htSKP‐EVs suggests that htSKP‐EVs not only promote the proliferation of hHFSCs but also induce the transition of hHFSCs to the anagen phase, thereby regulating the hair follicle cycle.

The hair follicle functions as a complex mini‐organ that depends on the dynamic interactions between epithelial and mesenchymal components.^49^ These bidirectional crosstalk events are crucial not only for the embryonic morphogenesis of hair follicles but also for regulating the progression of the postnatal hair cycle, including the transitions between telogen, anagen, and catagen phases.^50^, ^51^ SKPs, a multipotent mesenchymal precursor population in the hair follicle mesenchyme and closely associated with the dermal sheath and DPCs niche, serve as a central signaling hub that orchestrates this epithelial‐mesenchymal crosstalk.^52^ This interaction forms a stable tripartite regulatory loop with hDPCs and hHFSCs, supported by paracrine signaling (EVs as key effectors) and reciprocal feedback to sustain hair follicle homeostasis.

In regulating hDPCs, SKPs secrete EVs and soluble factors to preserve DPC trichogenicity (hair‐inductive potential) and aggregative growth capacity—hallmarks of functional DPCs that are lost during monolayer culture‐induced dedifferentiation.^53^, ^54^ While direct experimental evidence of SKP‐EVs modulating DPCs remains to be fully established, robust indirect evidence supports this mechanism: EVs from mesenchymal counterparts (e.g., MSCs, DPCs themselves) activate the Wnt/*β*‐catenin pathway in DPCs, deliver regulatory miRNAs (e.g., miR‐140‐5p and miR‐22‐5p), and enhance cell–cell adhesion to promote aggregation.^55^, ^56^, ^57^, ^58^ These effects are highly plausible for SKP‐EVs given their shared mesenchymal lineage and niche localization.^59^ Accordingly, htSKP‐EVs preserved the typical aggregative morphology of human DPCs without promoting scratch‐wound closure (Figure S6), consistent with the notion that SKP signals stabilize the adhesive, low‐migratory phenotype required for in situ trichogenic signaling rather than favoring dispersive migration. By sustaining DPC function, SKPs reinforce the DPC's role as the “command center” of hair follicle growth.

As a principal active component of EVs, miRNA, a type of non‐coding RNA, performs various regulatory functions across numerous biological processes.^60^, ^61^, ^62^, ^63^ miRNAs primarily affect cellular functions by modulating gene expression through two mechanisms: inhibiting mRNA translation and promoting degradation. The extent of base complementarity between the miRNA “seed” region and the target mRNA's 3′ UTR largely determines these outcomes. A high degree of complementarity induces mRNA degradation via RNA interference pathways, whereas a low degree of complementarity leads to the inhibition of mRNA translation.^64^ In this study, we utilized the Metascape and David databases to identify miR‐221‐3p as a target of the Wnt/*β*‐catenin pathway inhibitory protein DKK2, with each mRNA exhibiting a six‐nucleotide pairing site in the “seed” region. We further validated the targeting relationship between miR‐221‐3p and DKK2 using dual‐luciferase reporter assays in cells.

The Dkk (Dickkopf) protein family serves as a classic negative regulator of the Wnt/*β*‐catenin signaling pathway. In vertebrates, this family comprises four members, Dkk‐1 through Dkk‐4, with sizes ranging from 255 to 350 amino acids. Dkk‐1, Dkk‐2, and Dkk‐4 exhibit high structural similarity and contain two conserved cysteine‐rich domains that facilitate protein–protein interactions. DKK proteins not only bind to LRP5/6 but also act as high‐affinity ligands for the transmembrane proteins Kremen 1 and 2. The interaction among LRP6, DKK, and Kremen initiates the internalization and degradation of the LRP receptor, thereby inhibiting the Wnt/*β*‐catenin signaling pathway.^65^ A large‐scale meta‐analysis of genome‐wide association study data on male pattern baldness identified DKK2 as one of the most critical candidate genes likely implicated in the key pathophysiological features of male pattern baldness.^66^ This finding suggests that DKK2 may represent a promising target for the development of innovative therapeutic interventions. Furthermore, another study demonstrated that hDPCs can regulate the levels of Wnt/*β*‐catenin signaling in hair epithelial cells and in themselves through paracrine and autocrine DKK2, thereby sustaining the normal hair follicle cycle.^67^ Additionally, hairless areas in mice, such as the plantar skin, are primarily maintained by the specific high expression of DKK2, which inhibits the Wnt/*β*‐catenin signaling pathway; if DKK2 is knocked out, normal hair can develop on the plantar skin of the mouse.^68^ We speculate that miR‐221‐3p may activate the Wnt/*β*‐catenin signaling pathway by down‐regulating DKK2, which in turn promotes the proliferative activity of human dental pulp cells (hDPCs) and influences the hair follicle cycle. This hypothesis was validated in our experiments utilizing miR‐221‐3p mimics and inhibitors. Importantly, our previous findings suggest that human tissue‐derived stem cell extracellular vesicles (htSKP‐EVs) primarily enhance the proliferation rate of human hair follicle stem cells (hHFSCs), indicating that miR‐221‐3p is not the only factor through which htSKP‐EVs modulate the hair follicle cycle. Other components, including proteins and nucleic acids, also play significant roles and merit further investigation in future studies.

To investigate the influence of miR‐221‐3p on the biological functions of hair follicle cells, we developed a transfection vector for miR‐221‐3p. It is widely recognized that unmodified miRNAs exhibit instability and are highly vulnerable to degradation by the ubiquitous RNases present in the environment. The application of viral delivery systems is constrained by issues such as immunotoxicity, elevated mutation rates, and off‐target effects.^69^ Non‐viral delivery systems encompass naked oligonucleotides, chemically modified oligonucleotides, lipid carriers, dendrimers, and both natural and synthetic polymers, as well as EVs.^70^ miRNA‐mimics fall within this latter category; they are synthesized through chemical methods to replicate endogenous miRNAs, allowing them to enter cells when encapsulated in transfection reagents and temporarily enhance the activity of endogenous miRNAs. A recent study revealed that miRNA‐218‐5p is significantly upregulated in EVs derived from 3D cultured DPCs. Moreover, the injection of miRNA‐218‐5p‐mimic/polyethyleneimine (PEI) into the dorsal skin of mice has been demonstrated to promote hair follicle development.^71^ Consequently, for short‐term (<1 week) miRNA gain‐of‐function studies in cells, miRNA‐mimics represent a superior option.

This study underscores significant translational and industrial potential beyond mechanistic insights. First, htSKP‐EVs, sourced from discarded foreskin tissues that are abundant, ethically acceptable, and low‐cost, can be produced at scale through our optimized process while ensuring good biosafety. These EVs demonstrate promise as a novel cell‐free therapy for androgenetic and post‐traumatic alopecia, surpassing the efficacy of minoxidil. Second, our established preparation technologies facilitate the development of standardized kits for both research and clinical applications, while the elucidated mechanisms provide a theoretical foundation for cosmetic and aesthetic hair care products. Third, the miR‐221‐3p/DKK2/Wnt/*β*‐catenin axis presents biomarkers (miR‐221‐3p, DKK2) that can enhance hair loss diagnosis, efficacy monitoring, and clinical management. Finally, refining the interactions among SKPs, HFSCs, and DPCs will expedite the development of functional hair follicle organoids, thereby supporting high‐throughput drug screening, disease modeling, and translational research.

Despite the promising findings, this study has several limitations. First, we selected female mice to minimize experimental interference due to their low endogenous androgen levels, stable hair follicle cycles, and reduced aggressiveness, which decreases hair and skin damage from mutual biting. Future studies should include male models to validate cross‐gender applicability. Second, although we proposed a tripartite regulatory loop involving SKPs, DPCs, and HFSCs, the underlying mechanisms of their reciprocal regulation remain unclear. Additional co‐culture experiments, combined with multi‐omics analyses, are necessary to elucidate this interactive network. Third, given that EVs are complex mixtures, the synergistic effects of their proteins, lipids, and non‐coding RNAs, beyond miR‐221‐3p, on hair growth require further investigation. Fourth, a comprehensive long‐term and multi‐tier safety profile of htSKP‐EVs has yet to be established. Finally, the optimal administration route, whether topical or intradermal injection, as well as the dosage of htSKP‐EVs, necessitates further optimization.

In conclusion, htSKP‐EVs isolated via directed induction, culture transition, and gradient ultracentrifugation promote hair growth both in vitro and in vivo. Mechanistic studies further demonstrate that miR‐221‐3p downregulates DKK2 in dermal papilla cells, thereby activating the Wnt/*β*‐catenin signaling pathway and regulating hair follicle cycling. Collectively, we identify a novel miR‐221‐3p/DKK2/Wnt/*β*‐catenin axis that offers a mechanistic framework for DKK2‐driven alopecia pathogenesis. This discovery may open new avenues for identifying novel therapeutic targets and developing regenerative medicine‐based strategies for alopecia.

## AUTHOR CONTRIBUTIONS

Lingyun Zhao, Lidan Xiong, and Li Li led the study design and prepared the manuscript. Lingyun Zhao, Anqi Li, and Shijing Chen performed the experiments, analyzed the results, and Lingyun Zhao wrote a draft of the manuscript. Meng Hu, Nan Huang, Lingyu pan, Wei Hua and Runke Zhou discussed the experiments and analyzed the data. Lingyun Zhao and Yuanyuan Han interpreted the results; Lingyun Zhao, Ru Dai, Wei Hua, and Shijing Chen finalized the manuscript.

## CONFLICT OF INTEREST STATEMENT

All authors announce that there are no competing interests in publishing this article.

## Supporting information


**Figure S1.** Display of cell morphology under light microscope of hFBs and htSKPs (scale = 100 μm).
**Figure S2.** htSKPs possess stem cell properties, including the potential for osteogenic, adipogenic and myofibroblastic differentiation. (Scale = 100 μm).
**Figure S3.** The extracted DPCs were identified by immunofluorescence markers: *β*‐catenin and DAPI. The upper image shows *β*‐catenin (green fluorescence) nuclear DAPI staining (blue fluorescence), with expression in the cell membrane and cytoplasm of *β*‐catenin cells; the lower image shows ALP (red fluorescence) nuclear DAPI staining (blue fluorescence), with higher expression in the cytoplasm of ALP cells. *β*‐catenin and ALP are both specific markers for human DPCs, indicating that the cells isolated and cultured from the hair bulb tissue are indeed human DPCs. (DPCs: dermal papilla cells; DAPI: 4′,6‐Diamidino‐2‐phenylindole; ALP: Alkaline Phosphatase).
**Figure S4.** The microscopic morphology of hHFSCs after co‐culture with htSKP‐EVs.
**Figure S5.** Immunohistochemical images and statistical results of KRT14 (scale = 50 μm).
**Figure S6.** The invasiveness and migration of DPCs in htSKP‐EVs and hFB‐EVs group. In order to observe the effect of htSKP‐EVs on the migration ability of human DPCs, we conducted DPC scratch tests using the high‐concentration hFB‐EVs and htSKP‐EVs groups, and observed at 24, 48, and 72 h, as shown in Figure S5. The results revealed that the cell gaps did not show significant changes compared to before after 72 h, indicating that both types of extracellular vesicles had no significant impact on the migration ability of DPCs. (Scale = 200 μm, DPCs: dermal papilla cells; htSKP‐EVs: extracellular vesicles of transformed skin‐derived precursors; hFB‐EVs: human fibroblast‐derived extracellular vesicles).
**Figure S7.** Hematoxylin and eosin staining images of hair follicles in vitro.
**Figure S8.** Immunohistochemical images and statistical results of *β*‐catenin.
**Figure S9.** Immunohistochemical images and statistical results of Wnt10b.
**Figure S10.** Immunohistochemical images and statistical results of LEF1.
**Figure S11.** After subcutaneous injection at 0, 24, 48, 72, and 96 h, the biodistribution of htSKP‐EVs (CM‐Dil marked) in the skin, heart, liver, spleen, lung, kidney and brain via IVIS. 24 h post‐injection, a strong fluorescent signal from htSKP‐EVs was first detected in the liver, while a weaker signal was observed in the lungs. Fluorescence was not detected in other organs. Additionally, the fluorescence in the liver remained at a high intensity level 48 h after injection. From these observations, it can be inferred that the primary metabolic processes of htSKP‐EVs occur in the liver, which was in line with the main retention and clearance mechanisms of EVs. (IVIS: in vivo imaging system; htSKP‐EVs: extracellular vesicles of transformed skin‐derived precursors; hFB‐EVs: human fibroblast‐derived extracellular vesicles).
**Figure S12.** Dose–response in vivo experiments for htSKP‐EVs, photographs of hair growth status for each group. (*n* = 3, Control: equal amount of PBS injection, Low‐dose: 1 × 10^8^ particles/mL in 0.1 mL PBS, Mid‐dose: 1 × 10^9^ particles/ml in 0.1 mL PBS, High‐dose: 1 × 10^10^ particles/mL in 0.1 mL PBS, nine‐point subcutaneous injection, htSKP‐EVs: extracellular vesicles of transformed skin‐derived precursors).
**Figure S13.** Further HE staining of the high‐dose group tissue sections of each organ revealed no pathological changes. (scale = 50 μm).
**Figure S14.** Hematoxylin and eosin staining images of the mice alopecia model in each group.
**Figure S15.** Immunohistochemical images and statistical results of *β*‐catenin.
**Figure S16.** Immunohistochemical images and statistical results of LEF1.
**Figure S17.** Immunohistochemical images and statistical results of TCF3.
**Figure S18.** Immunohistochemical images and statistical results of Wnt3a.
**Figure S19.** Go annotation results of ht‐SKPs‐EV proteins.
**Figure S20.** COG annotation results of htSKP‐EVs proteins.
**Figure S21.** miRNA length distribution.
**Figure S22.** The transfection efficiency of miR‐221‐3p‐mimic on human DPCs was found to be the highest at 60 nM.
**Figure S23.** H&E staining images of different treatment groups.
**Figure S24.** Immunohistochemical images and statistical results of DKK2.
**Figure S25.** Immunohistochemical images and statistical results of β‐catenin.
**Figure S26.** Immunohistochemical images and statistical results of LEF1.
**Figure S27.** Immunohistochemical images and statistical results of C‐myc.
**Figure S28.** Immunohistochemical images and statistical results of CyclinD1.
**Figure S29.** Hematoxylin and eosin staining images of multiple organs in agomir group at 28 days.
**Table S1:** Detailed information of primer sequence.
**Table S2:** Small‐RNA sequencing quality control and read annotation summary for htSKP‐EVs and hFB‐EVs.

## Data Availability

The data that support the findings of this study are openly available in miRNA sequencing/proteomics for ht‐SKPs‐EV and FBs‐EV at https://www.omicsdi.org/dataset/project/PRJNA1194988, reference number PRJNA1194988 and PXD058677.
