# Supplementary material for: Extracellular vesicles of human transformed skin‐derived precursors containing miR‐221‐3p promote hair growth through DKK2‐mediated Wnt/ β ‐catenin signaling
Source: Bioeng Transl Med. 2026 Mar 30;11(3):e70130. doi: 10.1002/btm2.70130 (PMC13247428; doi:10.1002/btm2.70130)
Supplement: Supplementary file 1 — Figure S1. Display of cell morphology under light microscope of hFBs and htSKPs (scale = 100 μm). Figure S2. htSKPs possess stem cell properties, including the potential for osteogenic, adipogenic and myofibroblastic differentiation. (Scale = 100 μm). Figure S3. The extracted DPCs were identified by immunofluorescence markers: β‐catenin and DAPI. The upper image shows β‐catenin (green fluorescence) nuclear DAPI staining (blue fluorescence), with expression in the cell membrane and cytoplasm of β‐catenin cells; the lower image shows ALP (red fluorescence) nuclear DAPI staining (blue fluorescence), with higher expression in the cytoplasm of ALP cells. β‐catenin and ALP are both specific markers for human DPCs, indicating that the cells isolated and cultured from the hair bulb tissue are indeed human DPCs. (DPCs: dermal papilla cells; DAPI: 4′,6‐Diamidino‐2‐phenylindole; ALP: Alkaline Phosphatase). Figure S4. The microscopic morphology of hHFSCs after co‐culture with htSKP‐EVs. Figure S5. Immunohistochemical images and statistical results of KRT14 (scale = 50 μm). Figure S6. The invasiveness and migration of DPCs in htSKP‐EVs and hFB‐EVs group. In order to observe the effect of htSKP‐EVs on the migration ability of human DPCs, we conducted DPC scratch tests using the high‐concentration hFB‐EVs and htSKP‐EVs groups, and observed at 24, 48, and 72 h, as shown in Figure S5. The results revealed that the cell gaps did not show significant changes compared to before after 72 h, indicating that both types of extracellular vesicles had no significant impact on the migration ability of DPCs. (Scale = 200 μm, DPCs: dermal papilla cells; htSKP‐EVs: extracellular vesicles of transformed skin‐derived precursors; hFB‐EVs: human fibroblast‐derived extracellular vesicles). Figure S7. Hematoxylin and eosin staining images of hair follicles in vitro. Figure S8. Immunohistochemical images and statistical results of β‐catenin. Figure S9. Immunohistochemical images and statistical results [file BTM2-11-e70130-s001.docx]

**Supplemental Online Content**

**Title: Extracellular vesicles of human transformed** **skin-derived precursors containing miR-221-3p promote hair growth through DKK2-mediated Wnt/β-catenin signaling**

1. **eFigure section**


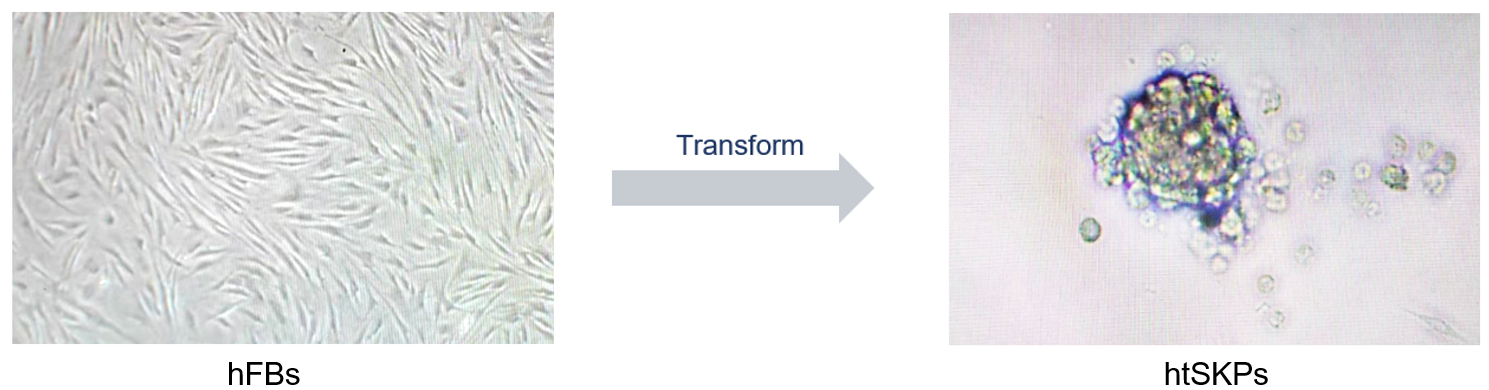


**Figure S1**. Display of cell morphology under light microscope of hFBs and htSKPs (scale = 100 μm). Under the light microscope, hFBs typically appear as flattened, irregularly shaped cells, and they have a large, oval nucleus with a prominent nucleolus. htSKPs in suspension culture appear as spherical or ovoid clusters, and the cells are tightly packed within the spheres, giving the appearance of a dense, cohesive structure. (hFBs: human fibroblasts; htSKPs: human transformed skin-derived precursors)


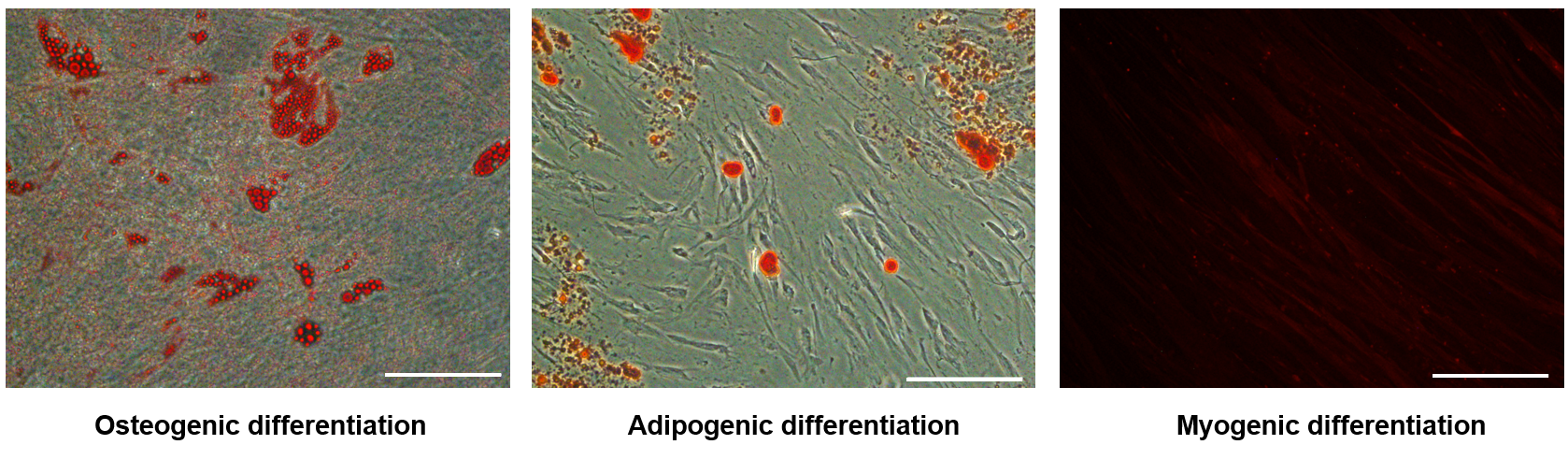


**Figure S2.** htSKPs possess stem cell properties, including the potential for osteogenic, adipogenic and myofibroblastic differentiation. (scale = 100 μm).

**Translation of the Tri-lineage Induction Process and Interpretation of Results for htSKPs**

**Osteogenic differentiation:** htSKPs were inoculated at a density of 2×10^4^ cells/ml in six-well plates. 2 ml of complete medium was added to each well. The plates were cultured in a 37°C, 5% CO2 incubator. The medium was changed every three days. When the cells reached 90%-100% of their adherent and fused state, the medium was replaced with osteogenic induction differentiation complete medium for osteogenic induction differentiation. The medium was changed every three days. The medium was the osteogenic induction differentiation complete medium. After induction for 20 days, the plates were rinsed three times with PBS. 1 ml of 4% paraformaldehyde solution was added to fix for 30 minutes. The plates were rinsed three times with PBS. 1 ml of safranin O staining solution was added to each well for 3-5 minutes, and then rinsed three times with PBS. The six-well plates were placed under a microscope for observation and photography. After culturing with the osteogenic induction solution for one week, the cell count could further increase. Subsequently, some cells aggregate and form high-density nodules. By day 20 of the culture, the central cells showed positive staining with calcein, indicating that they were calcium nodules, suggesting that htSKPs could undergo osteogenic differentiation. (Left side of Figure S2)

**Adipogenic differentiation:** After the htSKPs spheres were shaken into individual cells, they were inoculated into six-well plates at a density of 2-4×10^4^ cells/ml. Each well is added with 2 ml of complete medium. The cells are cultured in a 37°C, 5% CO2 incubator. The medium was changed every three days. When the ht-SKP cells reach 90%-100% of their adherent fusion, the medium was replaced with adipogenic induction differentiation medium A. After 3 days of induction, the adipogenic induction differentiation medium A was removed, and 2 ml of adipogenic induction differentiation medium B was added. One day later, the medium B was removed, and the medium A was replaced again for induction. This process of alternating between adipogenic induction differentiation medium A and B was repeated for 15 days. Finally, the medium A was continued to be used for culture for 5 days until large and round lipid droplets appear, indicating that adipogenic induction differentiation was complete. After adipogenic induction differentiation was completed, the medium is discarded, and the wells were washed three times with PBS. Each well was added with 1 ml of 4% paraformaldehyde solution for fixation for 30 minutes, and then washed three times with PBS. Subsequently, 30 minutes of oil red O staining agent was added to each well, and then rinsed three times with 1×PBS. The six-well plate was placed under a microscope for observation and photo-taking. After culturing with the lipogenic induction solution for 1 week, the cell morphology gradually transformed into a round-like shape. Two weeks later, some cells showed vacuolar changes and a stronger refractive property. After 20 days of cultivation, the vacuoles in the oil red-stained cells were brightly red, indicating that the htSKPs could differentiate into adipocytes (Central Figure S2).

**Myogenic differentiation:** htSKPs were seeded at a density of 2×10^4^ cells/ml in six-well plates. 2 ml of complete medium was added to each well and the plates were cultured in a 37°C, 5% CO2 incubator. The medium was changed every three days. When the cells reached 90%-100% of their adherent and fused state, the medium was replaced with smooth muscle induction differentiation medium. The medium was changed every three days. The induction medium was smooth muscle induction differentiation medium. After 20 days of induction, the medium was washed three times with PBS. 1 ml of 4% paraformaldehyde solution was added to each well for fixation for 30 minutes. The paraformaldehyde solution was removed and the wells were washed three times with PBS. Cell immunofluorescence staining was performed and the six-well plates were observed and photographed under a microscope. After being added to the smooth muscle differentiation culture medium, the cell morphology gradually became elongated and fusiform. After the induction process was completed, cell-specific immunofluorescence staining for the smooth muscle marker α-SMA was performed. A large number of red fluorescent fiber filaments could be observed, indicating that ht-SKPs have the ability to differentiate into smooth muscle (Right side of Figure S2)


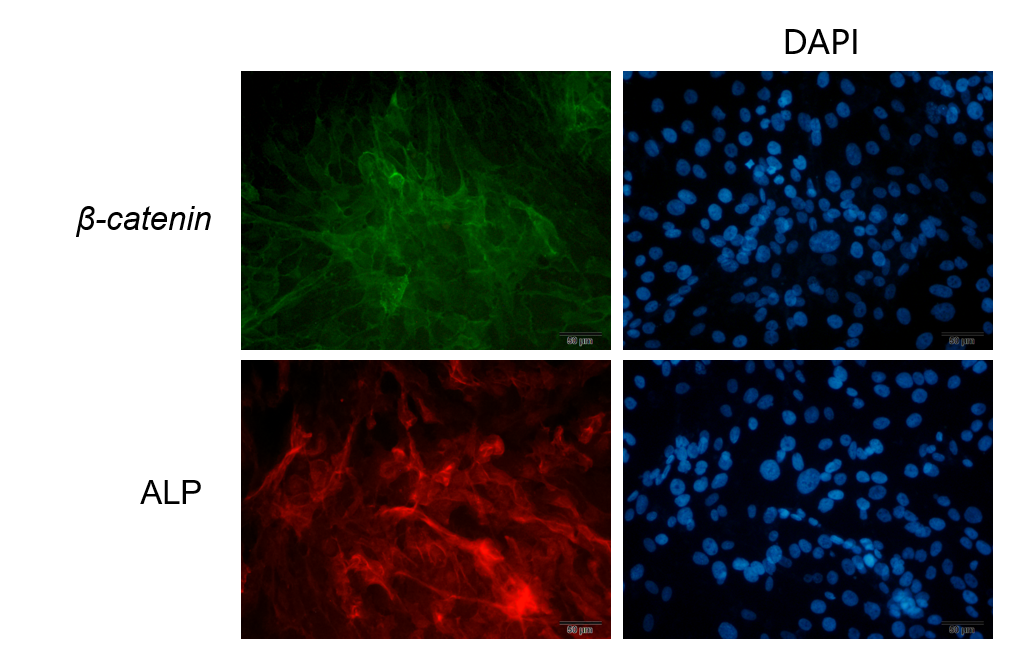


**Figure S3.** The extracted DPCs were identified by immunofluorescence markers: β-catenin and DAPI. The upper image shows β-catenin (green fluorescence) nuclear DAPI staining (blue fluorescence), with expression in the cell membrane and cytoplasm of β-catenin cells; the lower image shows ALP (red fluorescence) nuclear DAPI staining (blue fluorescence), with higher expression in the cytoplasm of ALP cells. β-catenin and ALP are both specific markers for human DPCs, indicating that the cells isolated and cultured from the hair bulb tissue are indeed human DPCs. (DPCs: dermal papilla cells; DAPI: 4',6-Diamidino-2-phenylindole; ALP: Alkaline Phosphatase)


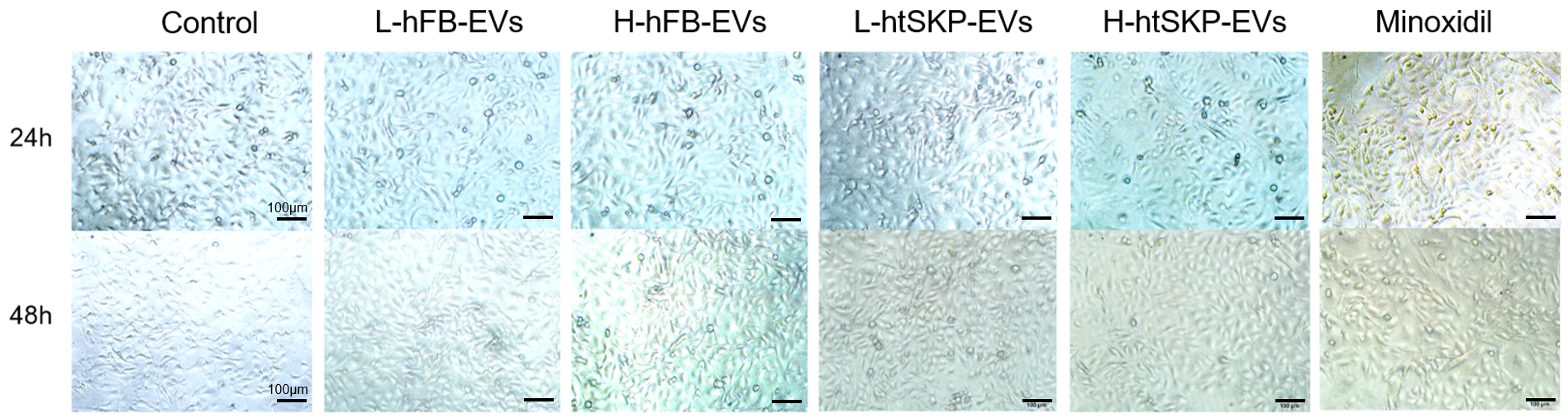


**Figure S4. The microscopic morphology of hHFSCs after co-culture with** **htSKP-EVs**

It shows the optical microscope images of HFSCs at different time points. Under light microscopy, HFSCs in htSKP-EVs groups appeared as small, cuboidal or polygonal cells with a high nuclear-to-cytoplasmic ratio. They had smooth surfaces and large nuclei with diffuse chromatin, indicating a high proliferative potential and active metabolic state. (scale = 100 μm, L: low concentration, 1×10^9 particles/ml, 96-well plate, 2 ul per well; H: high concentration, 1×10^10 particles/ml, 96-well plate, 2 ul per well; hHFSCs: human hair follicle stem cells; htSKP-EVs: extracellular vesicles of transformed skin-derived precursors).


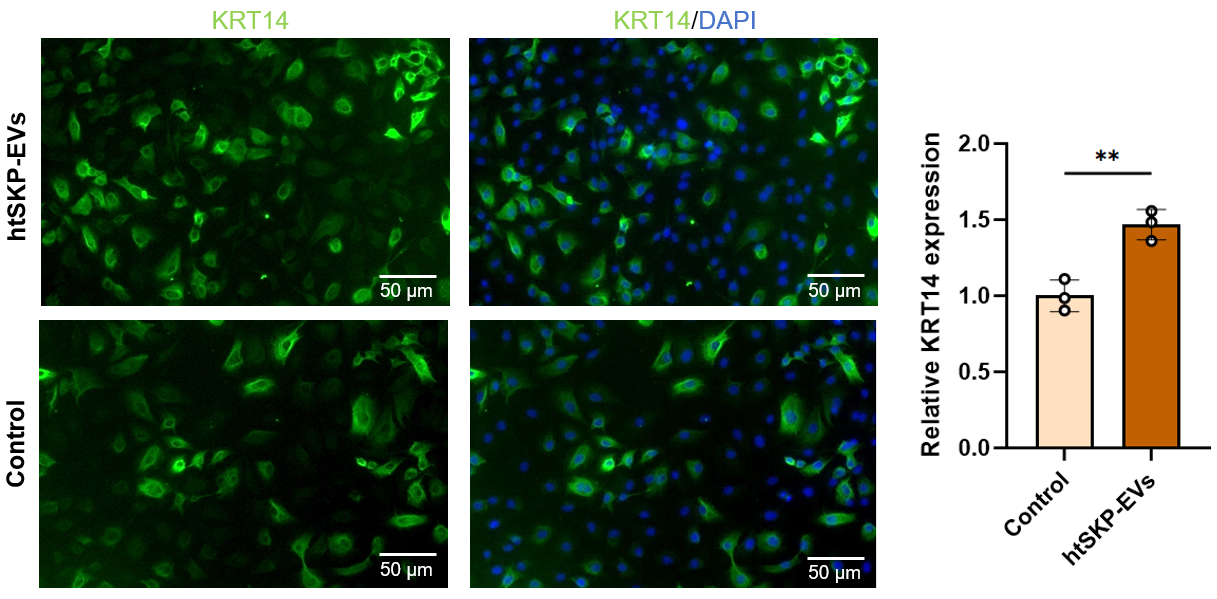


Figure S5. Immunohistochemical images and statistical results of KRT14 (scale = 50 μm).

To substantiate the proliferative state beyond bright-field morphology, we performed KRT14 immunofluorescence (basal-keratin marker of cycling HFSCs) combined with DAPI nuclear staining on hours-48 post-intervention. HFSCs were divided into blank control and htSKP-EVs intervention groups (1×10^10 particles/ml, 96-well plate, 2 ul per well). Representative images (new Figure S5) show that htSKP-EVs-treated cells exhibit significantly higher KRT14 expression (p < 0.01) and a larger proportion of small, oval nuclei typical of cycling cells, providing objective evidence that the htSKP-EVs group maintains an expanded, highly proliferative HFSC pool.


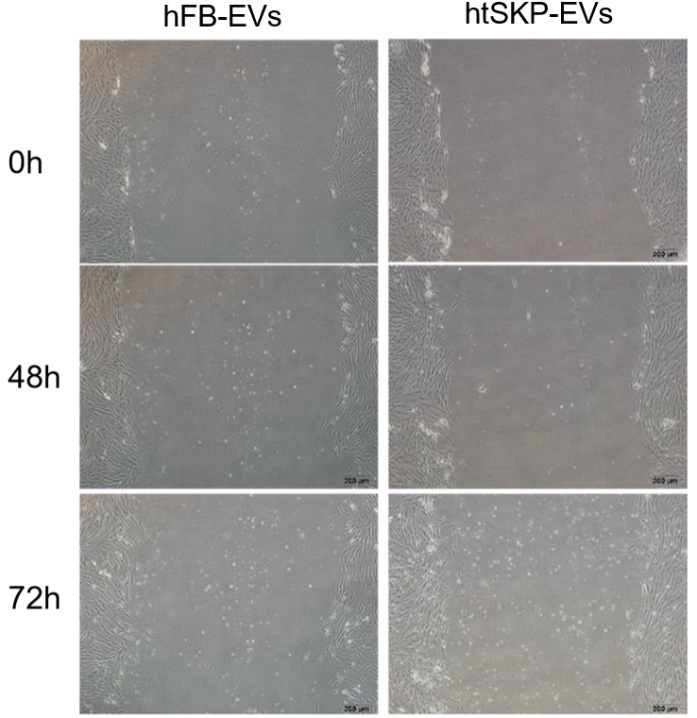


**Figure S6.** The invasiveness and migration of DPCs in htSKP-EVs and hFB-EVs group. In order to observe the effect of htSKP-EVs on the migration ability of human DPCs, we conducted DPC scratch tests using the high-concentration hFB-EVs and htSKP-EVs groups, and observed at 24 hours, 48 hours, and 72 hours, as shown in Figure S5. The results revealed that the cell gaps did not show significant changes compared to before after 72 hours, indicating that both types of extracellular vesicles had no significant impact on the migration ability of DPCs. (scale = 200 μm, DPCs: dermal papilla cells; htSKP-EVs: extracellular vesicles of transformed skin-derived precursors; hFB-EVs: human fibroblast-derived extracellular vesicles).


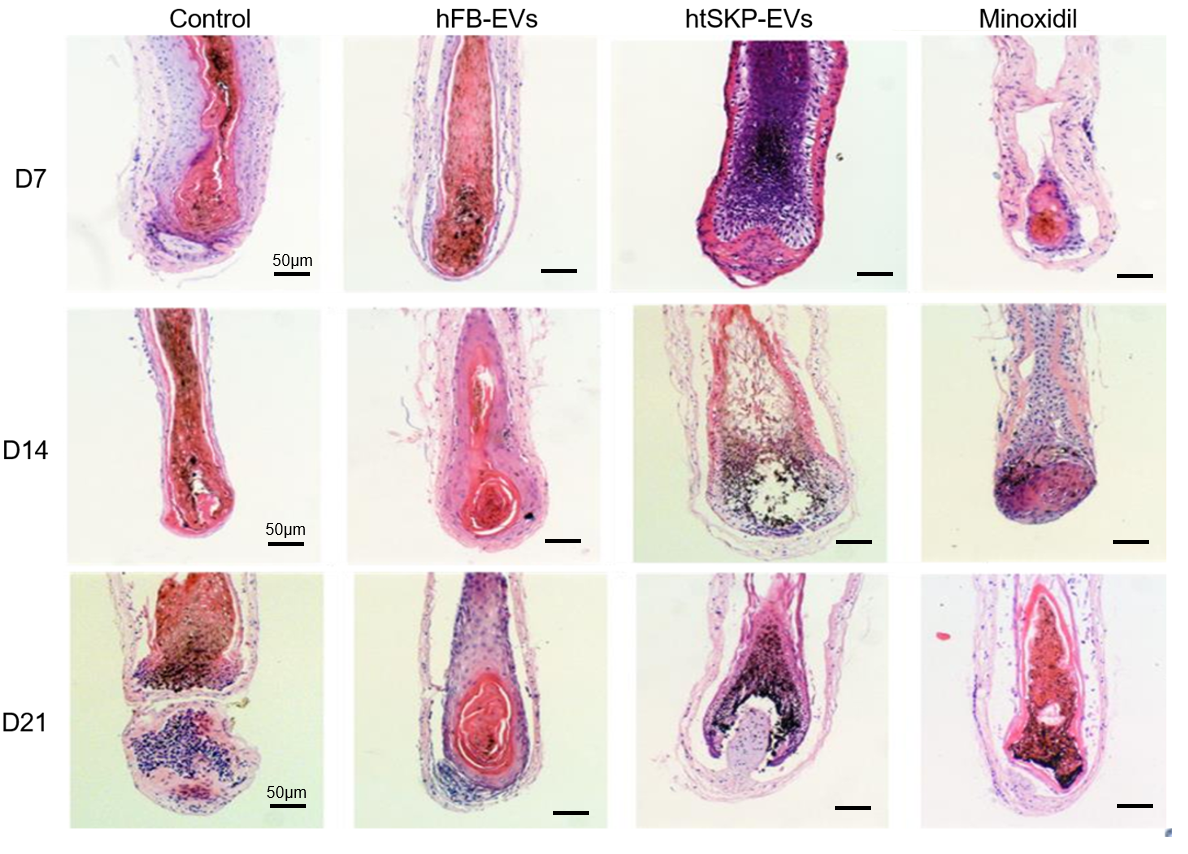


**Figure S7.** Hematoxylin and eosin staining images of hair follicles in vitro**.**

We conducted hematoxylin-eosin staining on the isolated hair follicles from different treatment groups on the 7th, 14th, and 21st days to observe the changes in the morphology of the isolated human hair follicles. The results are shown in Figure S6. On the 7th day, the isolated hair follicles from all groups showed degenerative changes, with the hair papilla becoming elliptical and the diameter of the hair papilla decreasing. On the 14th day, the isolated hair follicles in the blank group still showed degenerative changes, while those in the htSKP-EVs group began to expand, with abundant hair papilla matrix and an increased diameter, resembling an onion shape, indicating they were in the growth phase. The hFB-EVs group also showed similar changes, but the morphology was not as obvious as that of htSKP-EVs and hFB-EVs groups. The positive control group's hair papilla began to show growth-phase changes, but they were not as significant as those of htSKP-EVs and hFB-EVs groups. On the 21st day, the htSKP-EVs group remained in the growth-phase morphology, while the blank control group showed degenerative changes. (scale = 50 μm, htSKP-EVs: extracellular vesicles of transformed skin-derived precursors; hFB-EVs: human fibroblast-derived extracellular vesicles)


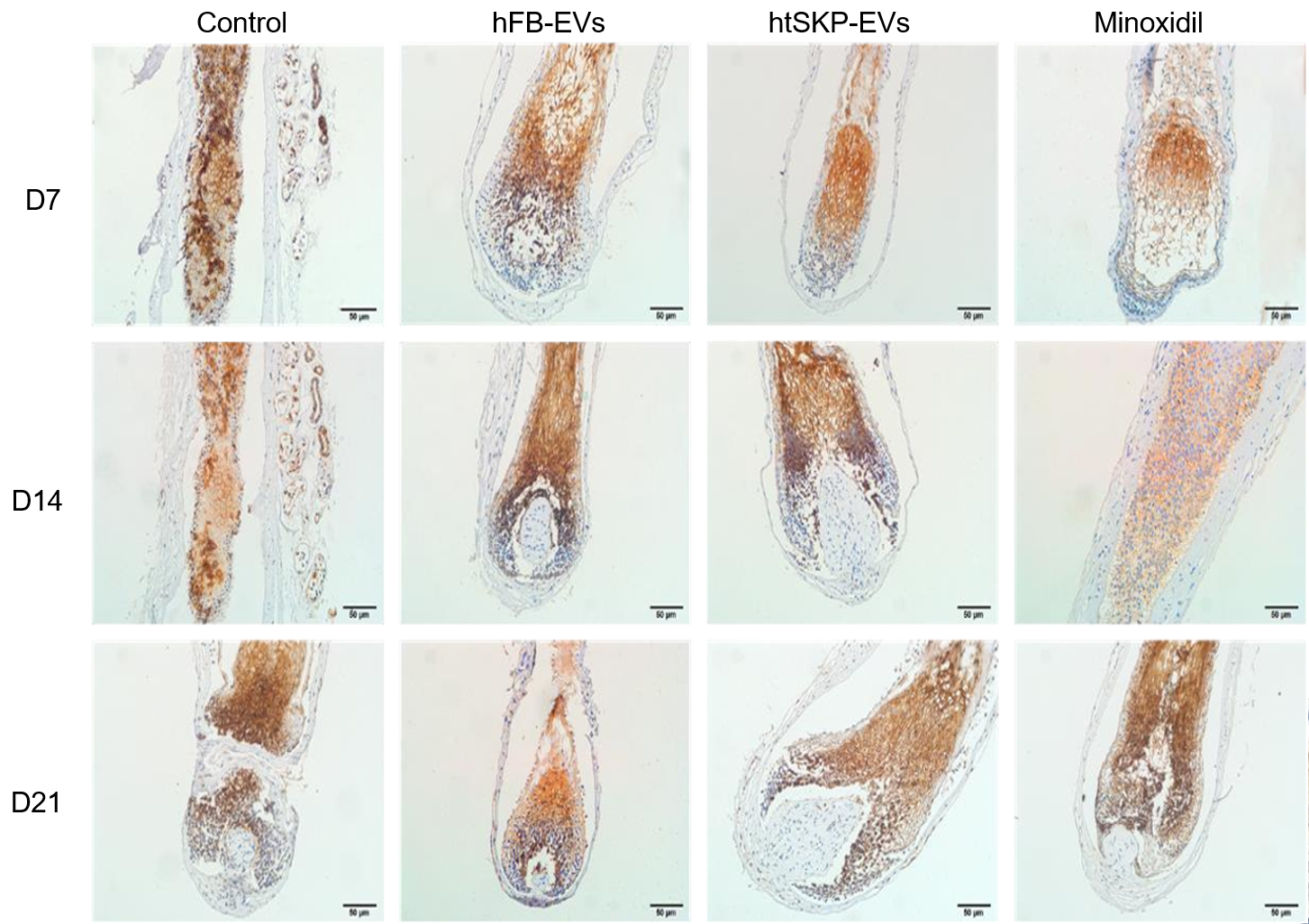


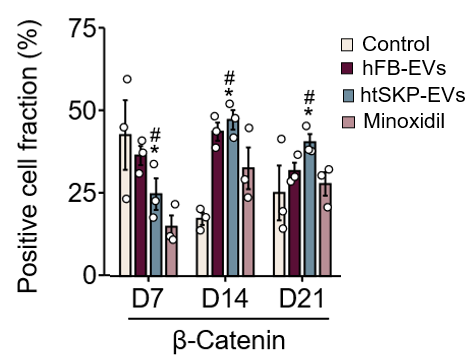


**Figure S8.** Immunohistochemical images and statistical results of β-catenin.

From top to bottom are the isolated hair follicles on the 7th day, 14th day, and 21st day. From left to right are the control group (only the same culture medium), the hFB-EVs group, the htSKP-EVs group, and the minoxidil group. The positive cells are mainly distributed in the hair matrix cells and the inner hair root sheath cells, while the expression of hair papilla cells is relatively low. The positive cell fraction of β-catenin was the highest in the htSKP-EVs group and the hFB-EVs group on the 14th day, and the value in the htSKP-EVs group was higher than that in the hFB-EVs group, and remained so until the 21st day. However, there was no statistically significant difference between the two groups. The positive fraction of β-catenin in the minoxidil group was lower than that in the htSKP-EVs group and the hFB-EVs group on the 7th day, the 14th day, and the 21st day (*p* < 0.05). The positive cell fraction of β-catenin in the blank control group was the highest on the 7th day, then decreased, and gradually increased again on the 21st day, showing no significant difference from the positive fraction of the minoxidil group (*p* > 0.05). This indicates that there is no significant difference in the promoting effect of β-catenin expression between htSKP-EVs and hFB-EV,s but both are stronger than that of minoxidil. (scale = 50 μm, mean ± SD; one-way ANOVA; * indicates *p* < 0.05 compared with the PBS control group; # indicates *p* < 0.05 compared with the Minoxidil group; + indicates *p* < 0.01 compared with the hFB-EVs group)htSKP-EVs: extracellular vesicles of transformed skin-derived precursors; hFB-EVs: human fibroblast-derived extracellular vesicles


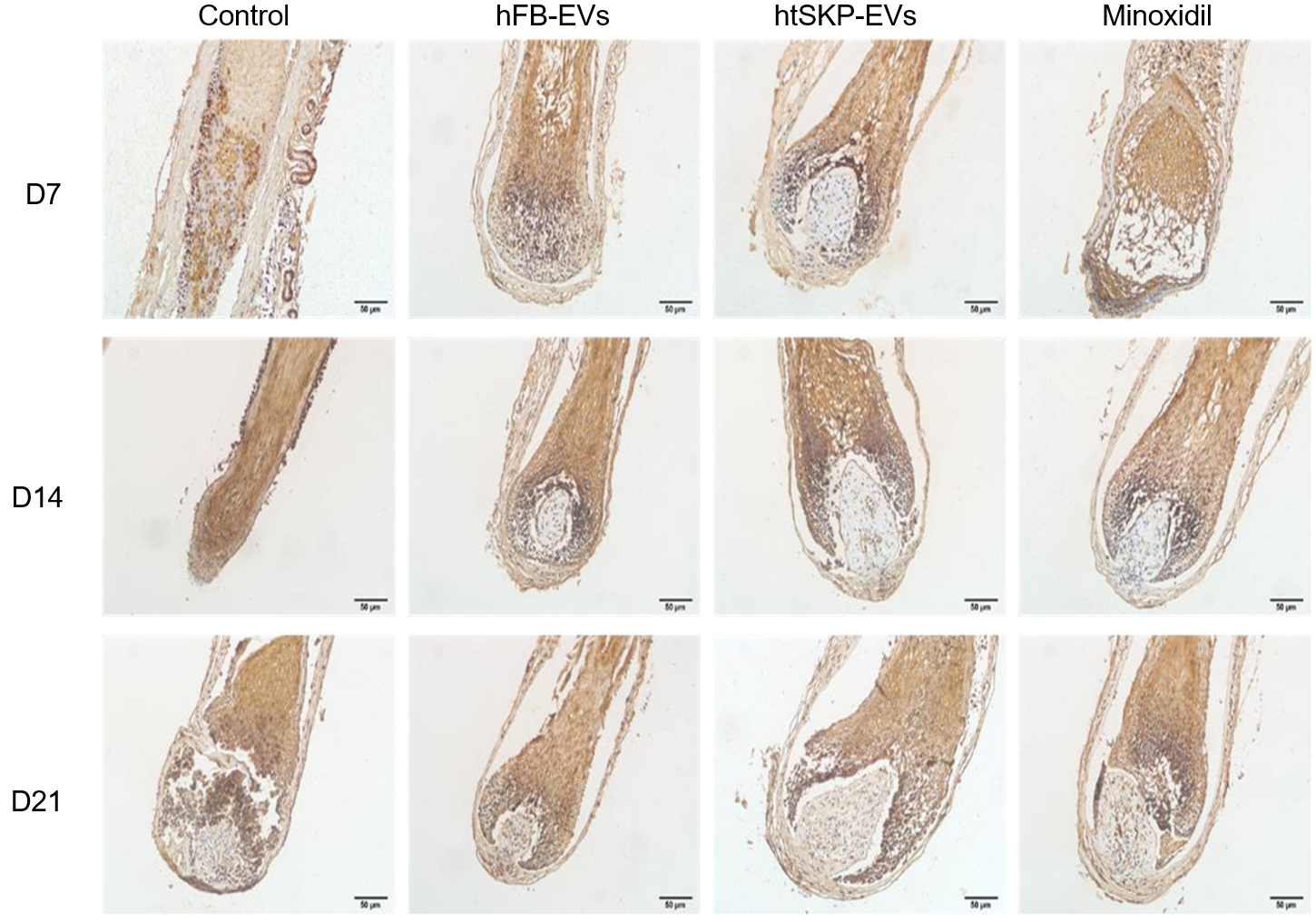


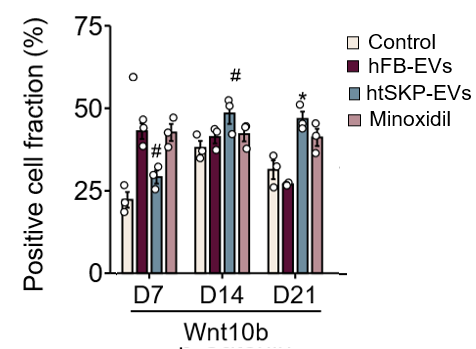


**Figure S9.** Immunohistochemical images and statistical results of Wnt10b.

The expression sites of Wnt10b are mainly concentrated in the outer root sheath, inner root sheath and hair matrix cells of the hair follicle, while the expression in hair papilla cells is relatively low. The positive cell fraction of Wnt10b in the htSKP-EVs group showed a trend consistent with that of β-catenin, reaching the highest level on the 14th day and persisting until the 21st day. The positive cell fraction of Wnt10b in the hFB-EVs group was higher on the 7th and 14th days, being 44.8 and 43.0 respectively, and decreased to 28.1 on the 21st day. Compared with htSKP-EVs, the difference was statistically significant (*p* < 0.05). The positive fraction of Wnt10b in the minoxidil group showed little change from the 7th to the 21st day, being 44.4, 43.9 and 42.9 respectively, indicating that minoxidil has no significant regulatory effect on Wnt10b. It is concluded that the regulatory effect of htSKP-EVs on Wnt10b was stronger than that of hFB-EVs and minoxidil. (scale = 50 μm, n = 3; mean ± SD; one-way ANOVA; * indicates *p* < 0.05 compared with the PBS control group; # indicates *p* < 0.05 compared with the Minoxidil group; + indicates *p* < 0.01 compared with the hFB-EVs group); htSKP-EVs: extracellular vesicles of transformed skin-derived precursors; hFB-EVs: human fibroblast-derived extracellular vesicles.


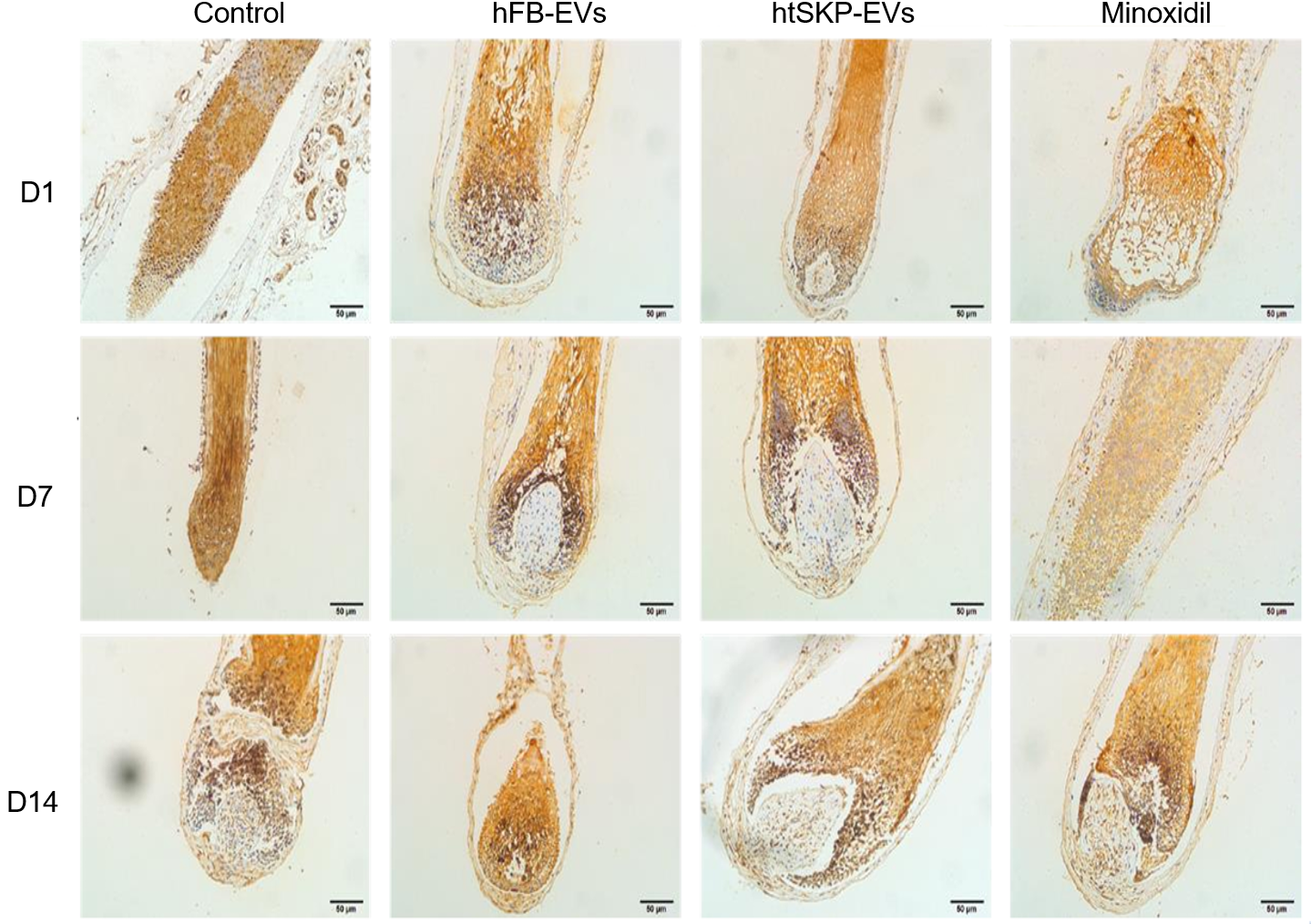


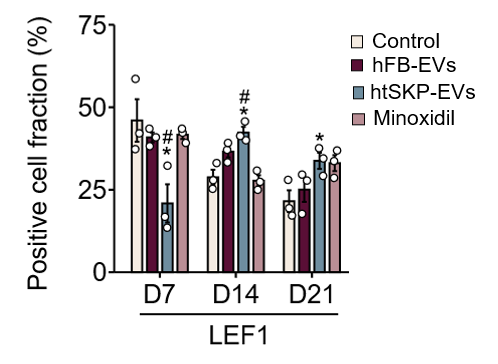


**Figure S10**. Immunohistochemical images and statistical results of LEF1.

From top to bottom, they are the isolated hair follicles on the 7th day, the 14th day, and the 21st day. From left to right, they are the control group, the hFB-EVs group, the htSKP-EVs group, and the minoxidil group. The expression sites of LEF1 are mainly concentrated in the outer hair root sheath, hair matrix cells, and inner root sheath cells. The expression in the hair papilla cells is also relatively abundant. In the htSKP-EVs group, the expression of LEF1 was lower on the 7th day. The positive score of LEF1 was the highest at 42.3 on the 14th day, which was higher than that of the hFB-EVs group (*p* > 0.05) and the minoxidil group (*p* < 0.05). On the 21st day, the scores of htSKP-EVs and the minoxidil group were still higher than those of the blank control group (*p* < 0.05), and there was no statistically significant difference between htSKP-EVs and the minoxidil group (*p* > 0.05). The trend of the positive score of LEF1 in the blank control group was consistent with that of β-catenin, reaching the highest on the 7th day and then decreasing. (scale = 50 μm; n = 3; mean ± SD; one-way ANOVA; * indicates *p* < 0.05 compared with the PBS control group; # indicates *p* < 0.05 compared with the Minoxidil group; + indicates *p* < 0.01 compared with the hFB-EVs group); htSKP-EVs: extracellular vesicles of transformed skin-derived precursors; hFB-EVs: human fibroblast-derived extracellular vesicles)


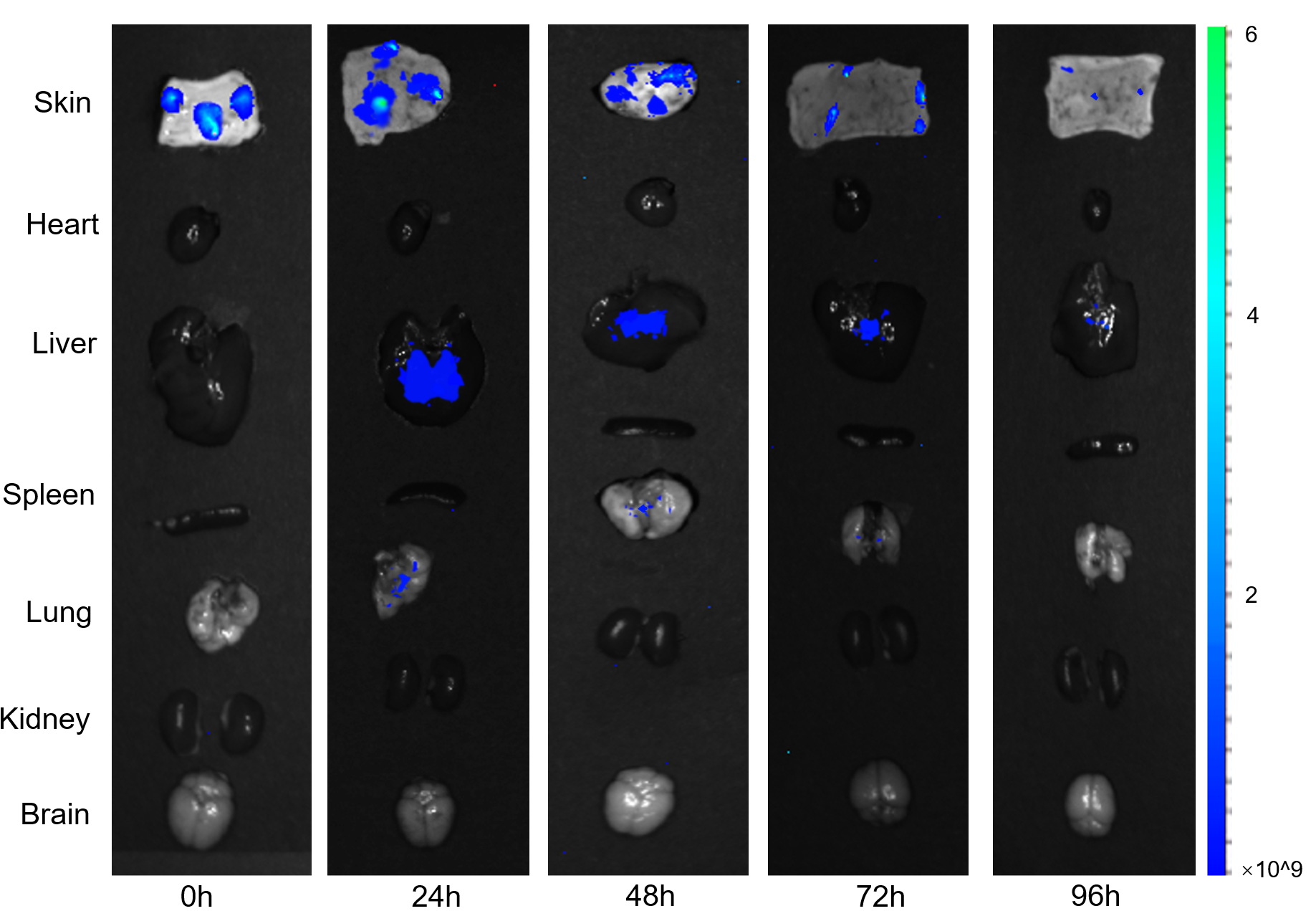


**Figure. S11.** After subcutaneous injection at 0h, 24h, 48h, 72h and 96h, the biodistribution of htSKP-EVs (CM-Dil marked) in the skin, heart, liver, spleen, lung, kidney and brain via IVIS. 24 hours post-injection, a strong fluorescent signal from htSKP-EVs was first detected in the liver, while a weaker signal was observed in the lungs. Fluorescence was not detected in other organs. Additionally, the fluorescence in the liver remained at a high intensity level 48 hours after injection. From these observations, it can be inferred that the primary metabolic processes of htSKP-EVs occur in the liver, which was in line with the main retention and clearance mechanisms of EVs. (IVIS: in vivo imaging system; htSKP-EVs: extracellular vesicles of transformed skin-derived precursors; hFB-EVs: human fibroblast-derived extracellular vesicles)


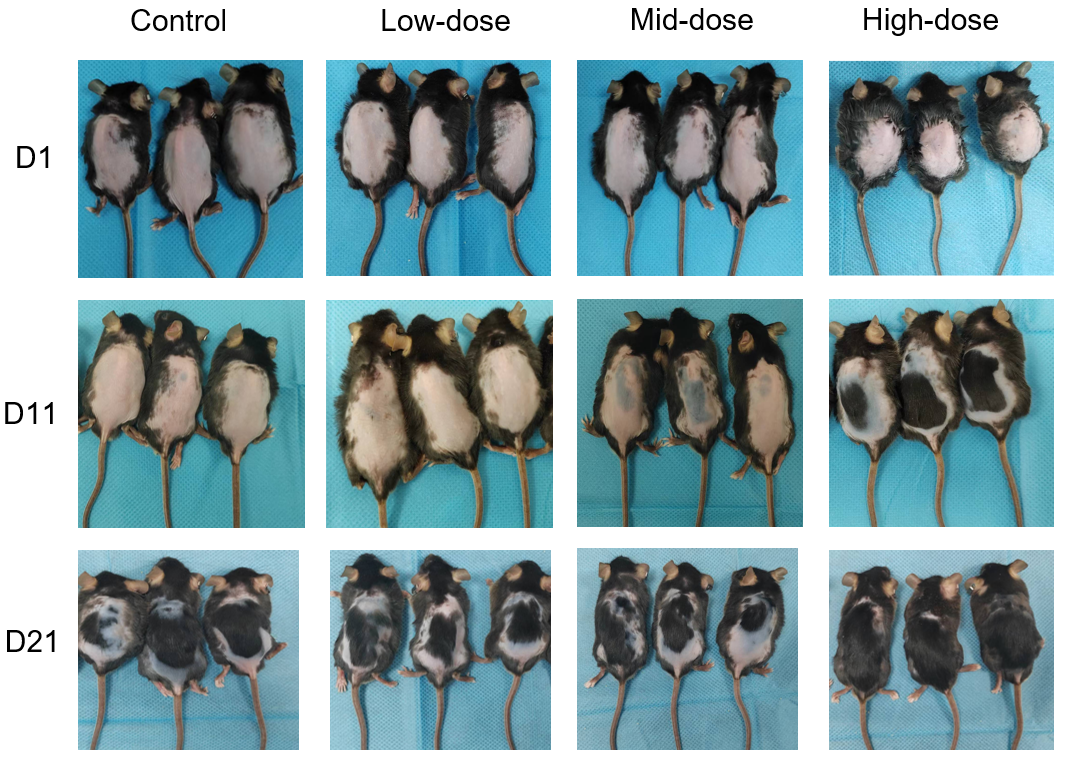


**Figure S12**. Dose-response in vivo experiments for htSKP-EVs, photographs of hair growth status for each group. (n=3, Control: equal amount of PBS injection, Low-dose: 1×10^8 particles/ml in 0.1ml PBS, Mid-dose: 1×10^9 particles/ml in 0.1ml PBS, High-dose: 1×10^10 particles/ml in 0.1ml PBS, nine-point subcutaneous injection, htSKP-EVs: extracellular vesicles of transformed skin-derived precursors)


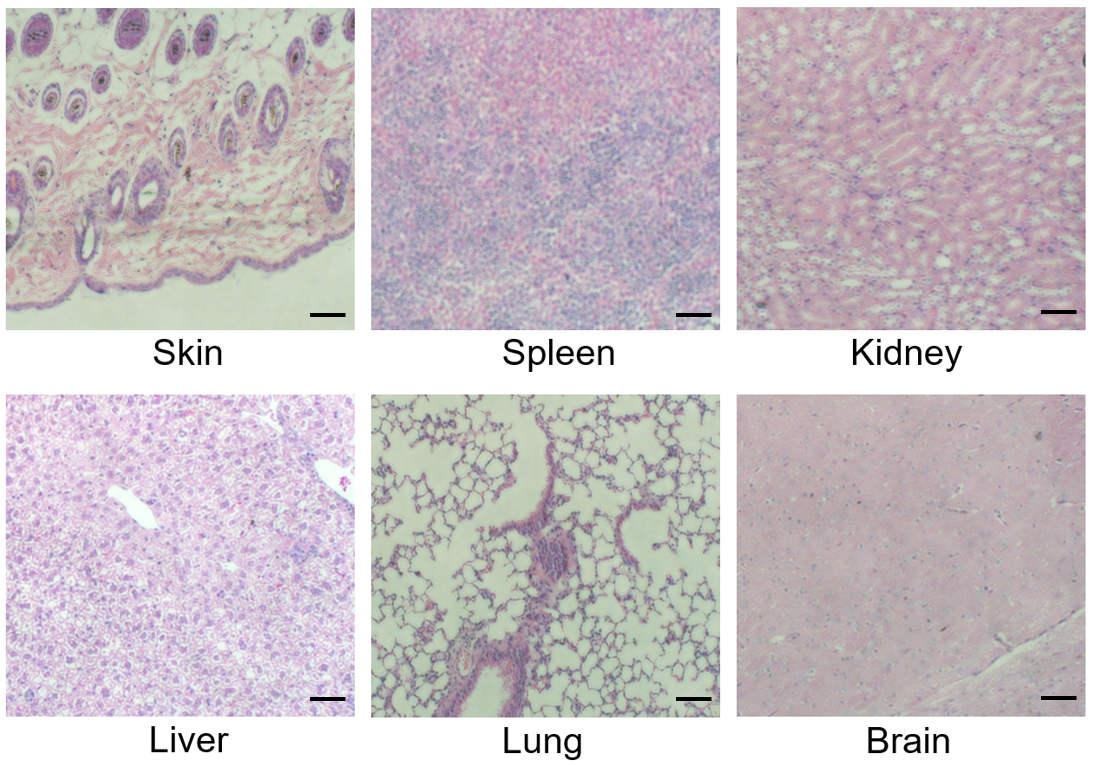


**Figure S13.** Further HE staining of the high-dose group tissue sections of each organ revealed no pathological changes. (scale = 50 μm).

**Dosage analysis and explanation:** In the mouse in vivo model, the injection volume of EVs was generally between 1×10⁸ – 3×10¹¹ particles per mouse per injection^1, 2, 3^. Based on body surface area conversion, for a mouse weighing 20 g, 0.1ml of intradermal injection corresponds to 0.5 mL/kg. According to the body surface area conversion method (FDA 2005), the conversion factor from 20 g of mouse to 60 kg of human ≈ 12.3. The equivalent dose for humans ≈ 0.5 mL/kg ÷ 12.3 ≈ 0.04 mL/kg, which means a single injection for a 60 kg adult ≈ 2.4 mL. The clinical hair regrowth test has safely used 0.5–1.0 mL/30–40 cm² of local injection on the scalp. Therefore, a dose of 1×10¹⁰ particles per mouse is only 2–3 mL on the human scalp to cover, which is completely within the clinical operational range. Meanwhile, in our research, the quantity of EVs in the high-dose group can be mass-produced and applied. Therefore, based on the experimental data in Figure S14 and the above explanations, we believe that the concentration (1×10^10 particles/ml, 0.1ml per mouse) we chose in the main text is reasonable.


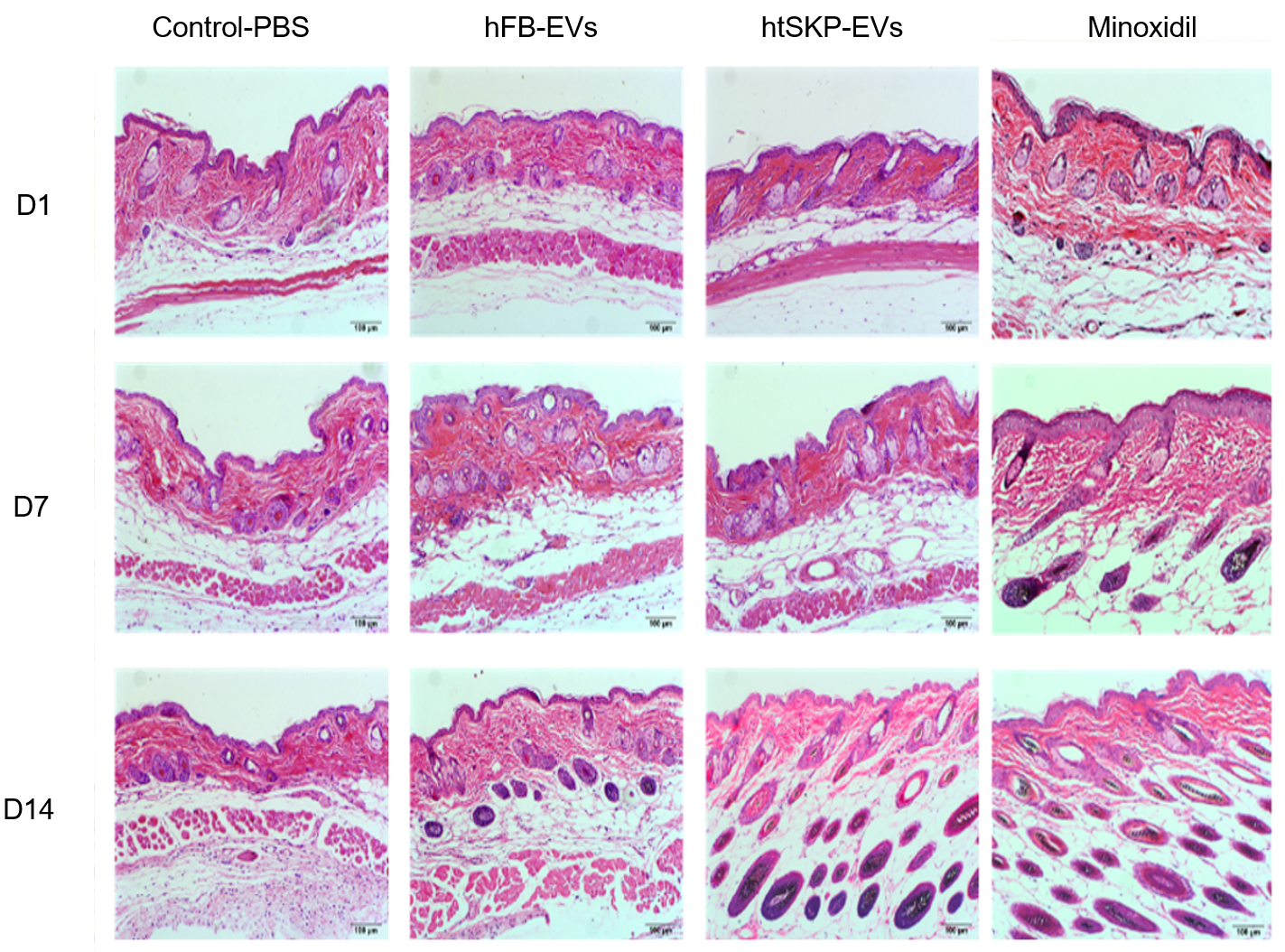


**Figure S14.** Hematoxylin and eosin staining images of the mice alopecia model in each group.

As can be found, with the intervention period increased, hair follicles became deeper and their numbers gradually increased. And as can be found from the figure, the htSKP-EVs and minoxidil groups showed the most significant results (scale = 50 μm, htSKP-EVs: extracellular vesicles of transformed skin-derived precursors; hFB-EVs: human fibroblast-derived extracellular vesicles; PBS: phosphate buffered saline)


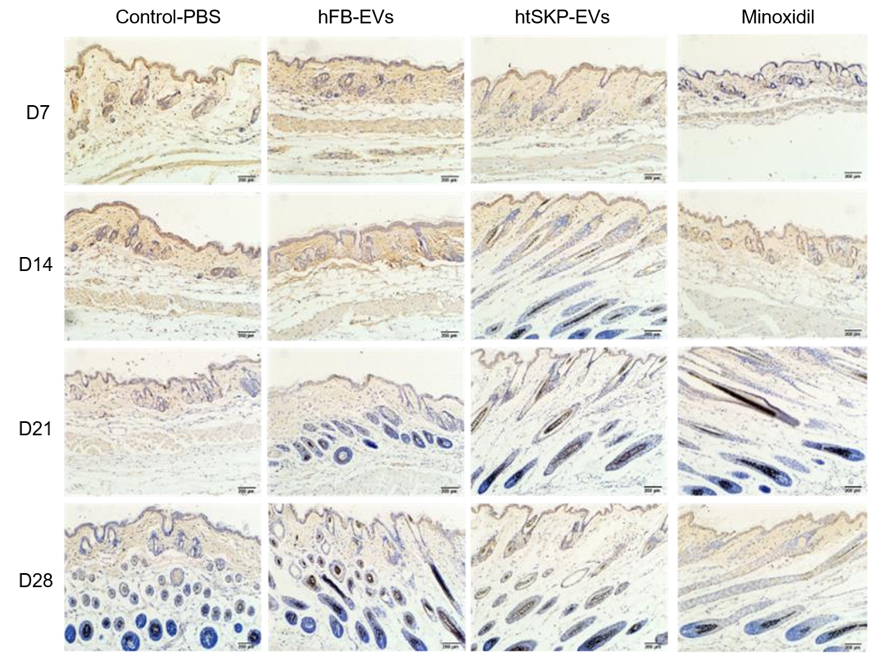


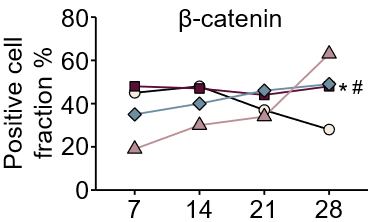


**Figure S15**. Immunohistochemical images and statistical results of β-catenin.

Different treatment groups showed certain levels of expression in epidermal and hair follicle outer hair root sheath cells. Among them, the expression levels of β-catenin in the htSKP-EVs group and minoxidil group increased with time. The positive cell fraction in the hFB-EVs group remained basically the same at the four time points. However, the expression of β-catenin in the blank control group decreased with time, and the difference was statistically significant (*p* < 0.05), indicating that htSKP-EVs and minoxidil all could promote the expression of β-catenin. On the 28th day, the positive cell fraction in the minoxidil group was the highest among all treatment groups, approximately 63. (scale = 50 μm; n = 3; mean ± SD; one-way ANOVA; * indicates *p* < 0.05 compared with the PBS control group; # indicates *p* < 0.05 compared with the Minoxidil group);htSKP-EVs: extracellular vesicles of transformed skin-derived precursors; hFB-EVs: human fibroblast-derived extracellular vesicles; PBS: phosphate buffered saline.


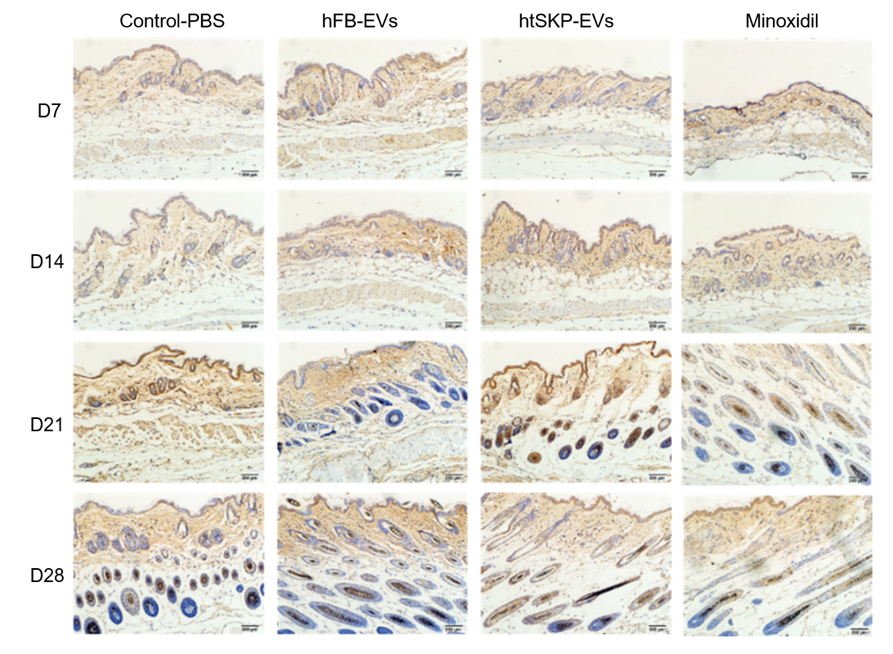


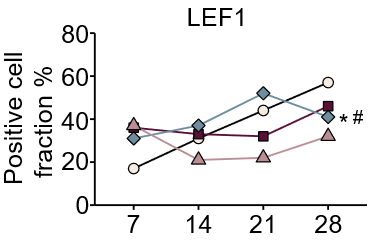


**Figure S16**. Immunohistochemical images and statistical results of LEF1.

LEF1 is expressed in the epidermal components of hair follicles and the nuclei of epidermal cells. In the PBS group, the positive cell fraction increases over time. The expression is highest on the 21st day in the htSKP-EVs decreased on the 28th day. The hFB-EVs group showed no significant difference in expression over the first 21 days, and reached the highest expression level on the 28th day. The minoxidil group had the highest expression on the 7th day, dropped significantly on the 14th day, and gradually increased again on the 28th day. The difference is statistically significant, *p* < 0.05. From this, it can be found that the LEF1 expression levels in the htSKP-EVs group increased the earliest, indicating the fastest effect of the regulation of the Wnt pathway. (scale = 50 μm; n = 3; mean ± SD; one-way ANOVA; * indicates *p* < 0.05 compared with the PBS control group; # indicates *p* < 0.05 compared with the Minoxidil group);LEF1: lymphoid enhancer-binding factor 1; htSKP-EVs: extracellular vesicles of transformed skin-derived precursors; hFB-EVs: human fibroblast-derived extracellular vesicles; PBS: phosphate buffered saline.


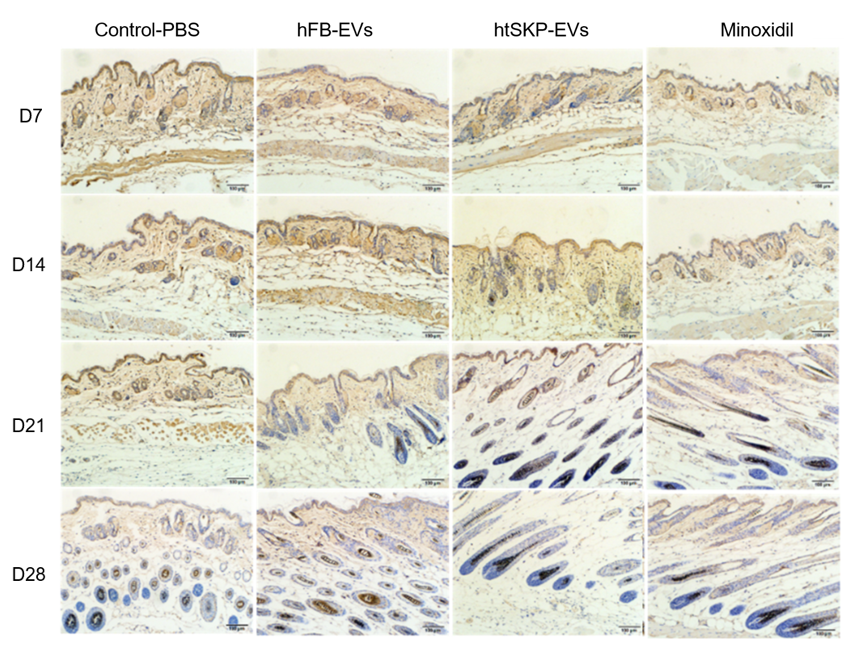


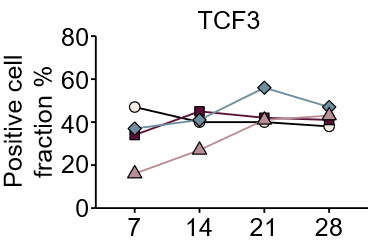


**Figure S17.** Immunohistochemical images and statistical results of TCF3.

In both the blank control group and the different treatment groups, there was a weak expression of TCF3 in the nuclei of hair follicle outer root sheath cells and epidermal cells. In the blank control group, the expression level of TCF3 did not change significantly over time; in the htSKP-EVs group, the expression level of TCF3 increased over time, reaching a peak on the 21st day, then decreased on the 28th day, which was consistent with the change trend of the minoxidil group; the expression level of LEF1 in the hFB-EVs group showed no significant change on the 14th, 21st, and 28th days, with *p* > 0.05. This indicates that the effect of htSKP-EVs on TCF3 expression is consistent with that of minoxidil, and it is the strongest on the 21st day. (scale = 50 μm; n = 3; mean ± SD; one-way ANOVA);htSKP-EVs: extracellular vesicles of transformed skin-derived precursors; hFB-EVs: human fibroblast-derived extracellular vesicles; TCF3: T-cell factor 3; PBS: phosphate buffered saline.


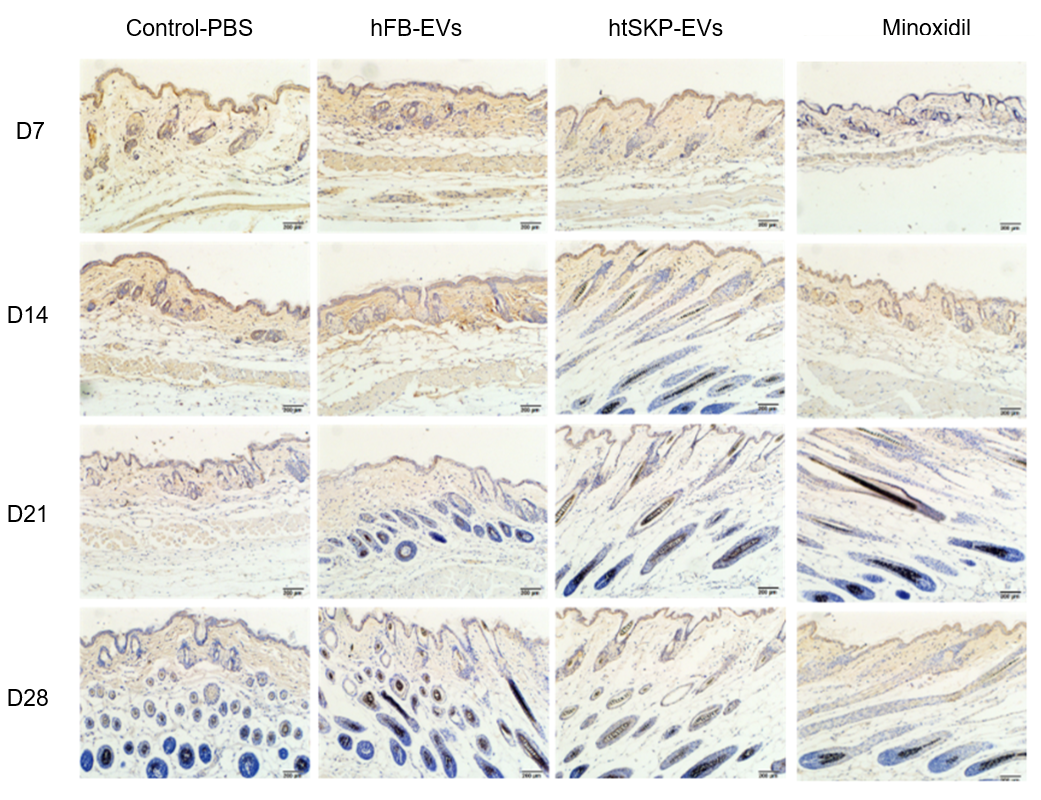


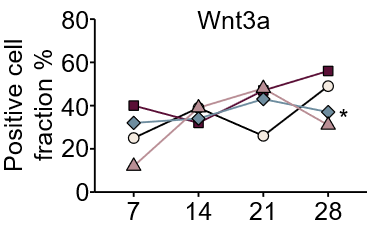


**Figure S18.** Immunohistochemical images and statistical results of Wnt3a.

The expression levels of the epidermal components and epidermal cells were at a relatively low level. On the 7th day, the expression level was the lowest in the minoxidil group, with a positive cell fraction of only 12. On the 14th day, there was no statistically significant difference in the expression levels of Wnt3a among the various groups (*p* > 0.05). On the 21st day, except for the blank control group, the expression levels of positive cells in the other groups increased. Among them, the htSKP-EVs group and minoxidil group reached their peak at the 21st day, with no significant difference in their expression levels. Then, they decreased on the 28th day. The peak of FB-EV occurred on the 28th day. This indicates that the effects of htSKP-EVs and minoxidil on the expression level of WNT3a occurred earlier than that of hFB-EVs. (scale = 50 μm; n = 3; mean ± SD; one-way ANOVA; * indicates *p* < 0.05 compared with the PBS control group);htSKP-EVs: extracellular vesicles of transformed skin-derived precursors; hFB-EVs: human fibroblast-derived extracellular vesicles; PBS: phosphate buffered saline.


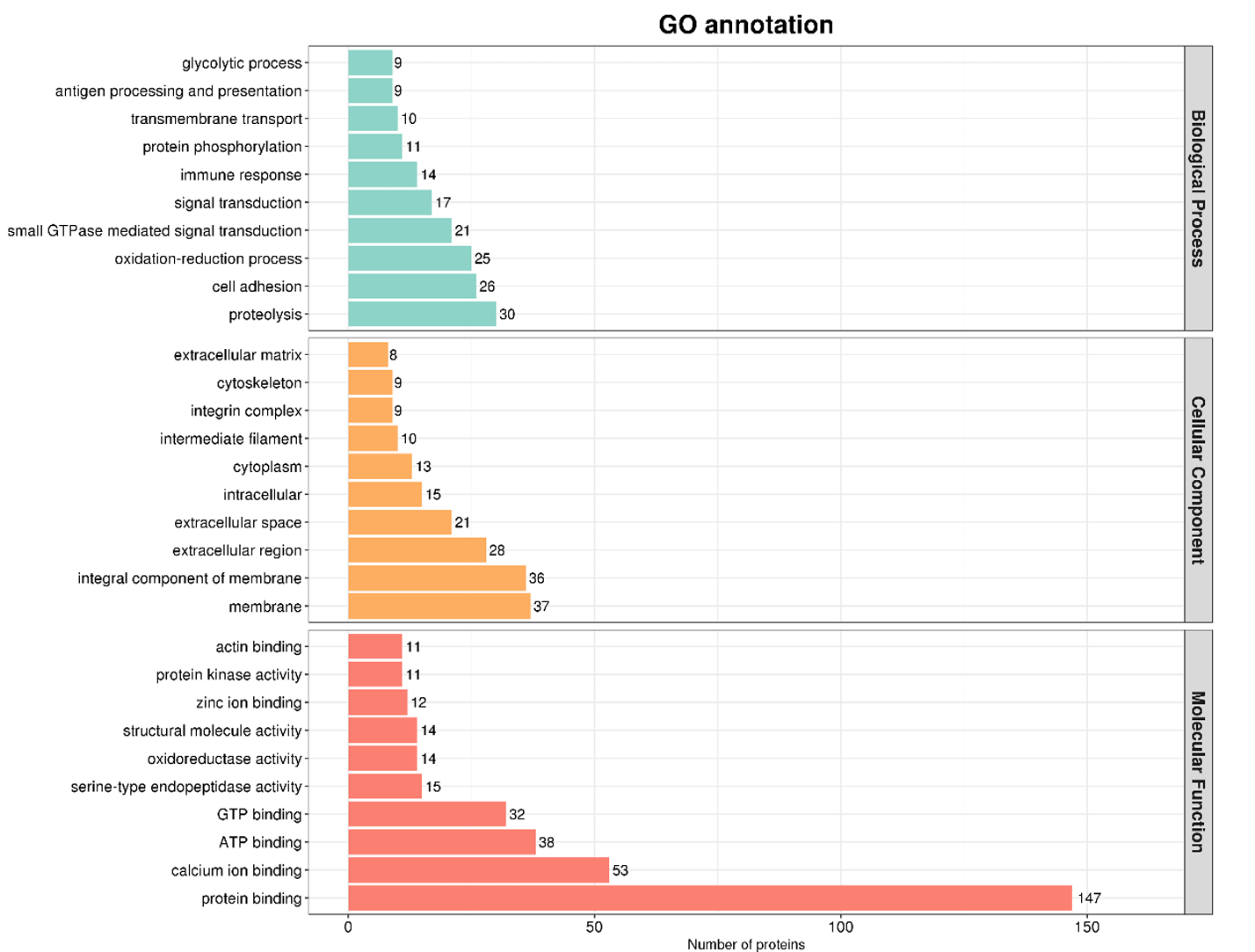


**Figure S19.** Go annotation results of ht-SKPs-EV proteins

The GO annotation results show that in terms of biological processes, the functions of the htSKP-EVs proteins mainly lie in proteolysis, cell adhesion, antioxidant activity, GTPase-related signal transduction, etc.; in terms of cellular components, the functions of the htSKP-EVs proteins mainly involve forming the cell membrane, intracellular membrane structures, and extracellular components, etc.; in terms of molecular functions, the htSKP-EVs proteins mainly function in protein binding, calcium ion binding, ATP binding, GTP binding, etc. (htSKP-EVs: extracellular vesicles of transformed skin-derived precursors)


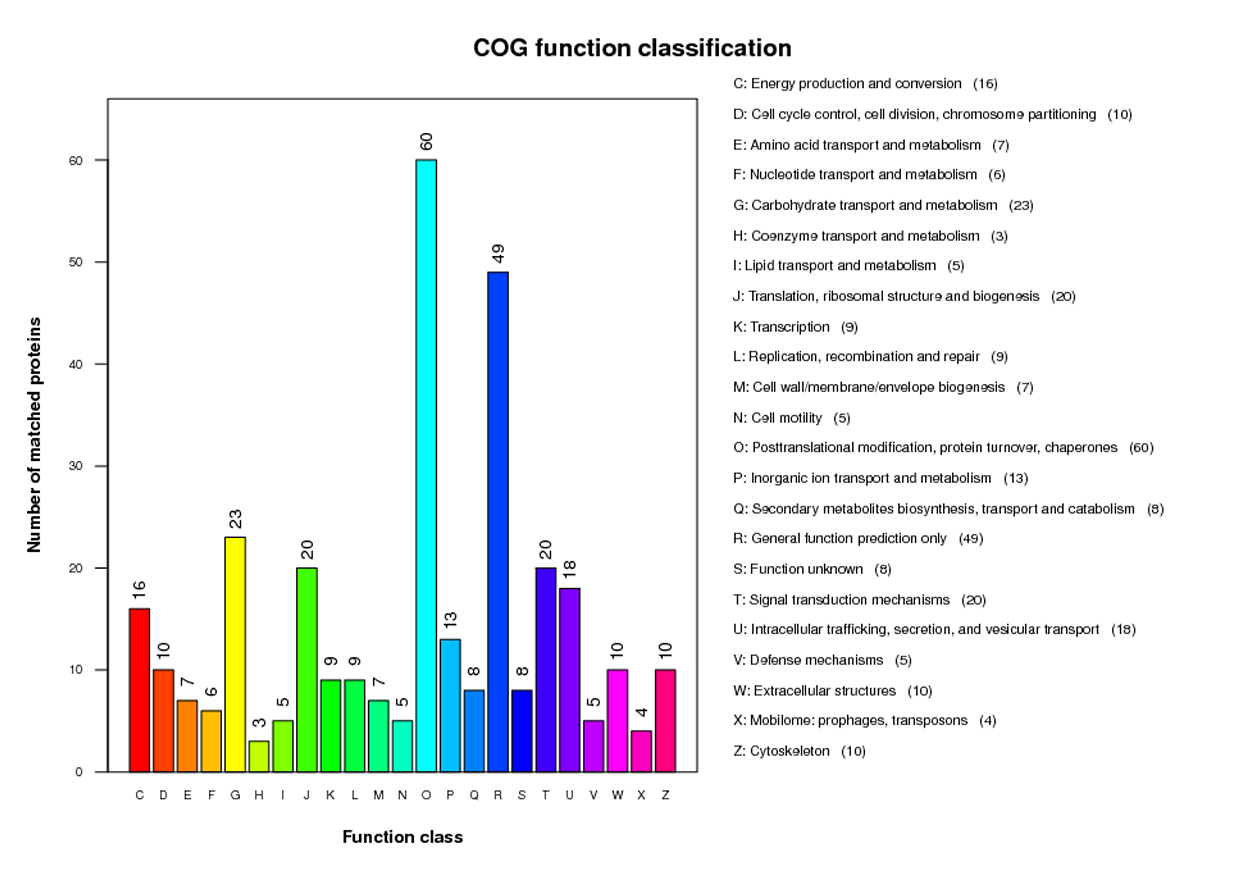


**Figure S20.** COG annotation results of htSKP-EVs proteins

The COG analysis results show that the functions of the htSKP-EVs protein are mainly concentrated in post-translational modifications, protein folding, molecular chaperones, and other protein modification functions. (htSKP-EVs: extracellular vesicles of transformed skin-derived precursors)


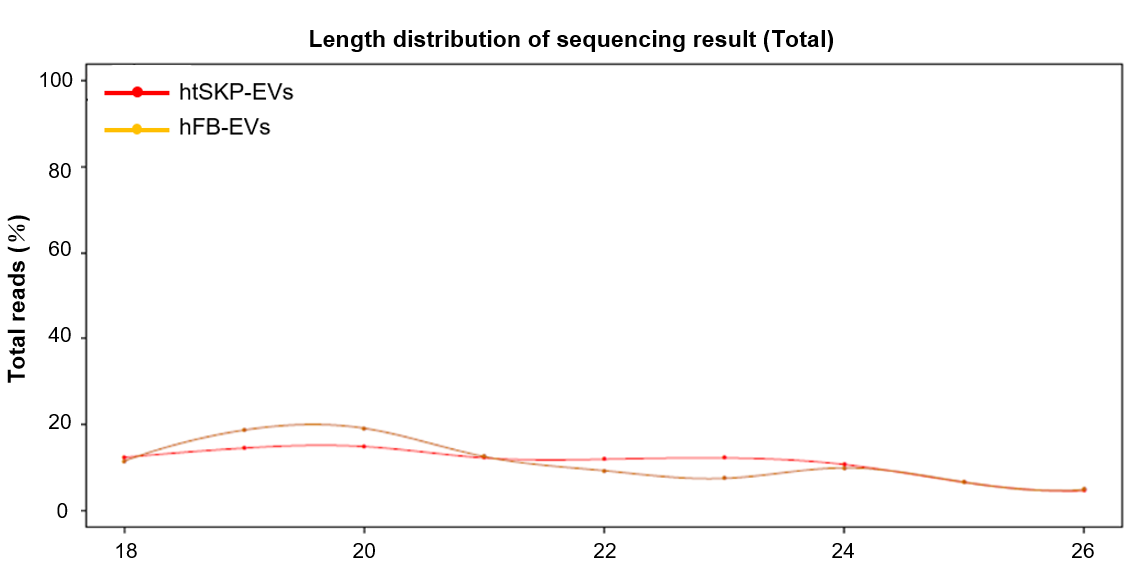


**Figure S21.** miRNA length distribution

As shown, the miRNA length distribution of both htSKP-EVs and hFB-EVs peaks at 18–26 nt, consistent with the canonical size range of mature miRNAs, confirming the sequencing quality and reliability of downstream analyses.


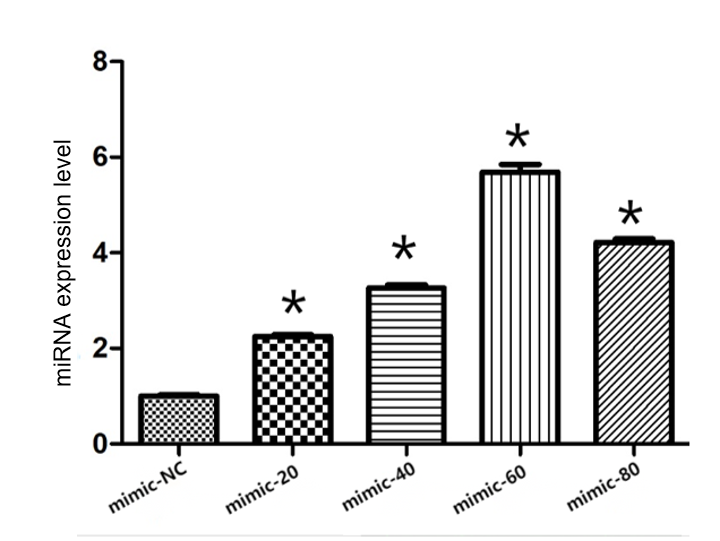


**Figure S22.** The transfection efficiency of miR-221-3p-mimic on human DPCs was found to be the highest at 60nM.


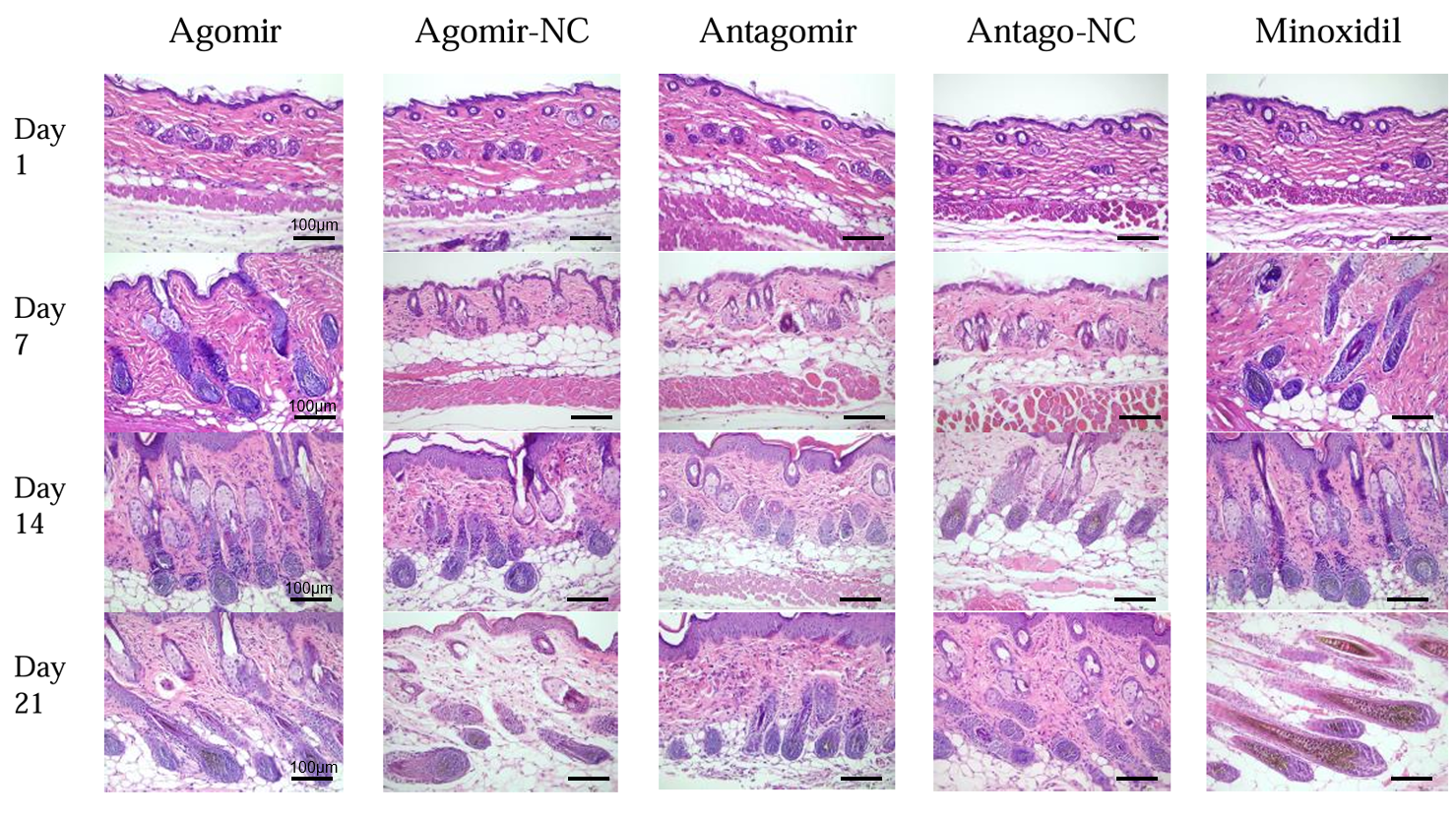


**Figure S23.** H&E staining images of different treatment groups.

Based on the results of H&E staining, we statistically analyzed the epidermal thickness, hair bulb diameter, and the number of hair follicles in different treatment groups of mice. Number and location of hair follicles: On the first day, the number of hair follicles in all treatment groups was relatively small, and the follicles were shallow, located in the deep dermis, and not reaching the subcutaneous fat layer. On the 7th day, a small number of hair follicles in the Agomir group and the Minoxidil group reached the subcutaneous fat layer, but the number of hair follicles in all groups was still small. On the 14th day, the number of hair follicles in the Agomir group and the Minoxidil group significantly increased, and more hair follicles reached the subcutaneous fat layer; on the 21st day, hair follicles in all groups reached the fat layer, but the Agomir group and the Minoxidil group were still significantly more than the other groups; Changes in epidermal thickness: The epidermal thickness of all groups gradually increased over time, with the greatest difference observed from the 7th to 14th day, and the change in epidermal thickness was not significant from the 14th to 21st day; Hair bulb diameter: As time passed, the hair follicles in all groups gradually showed characteristics of the growth phase, and the hair bulbs gradually expanded. Among them, the hair bulbs in the Agomir group and the Minoxidil group grew the fastest, starting to widen the gap from the 7th day compared to the other groups, indicating that both promoted the transformation of the hair follicle growth phase in mice. (scale = 100µm, H&E: hematoxylin and eosin)


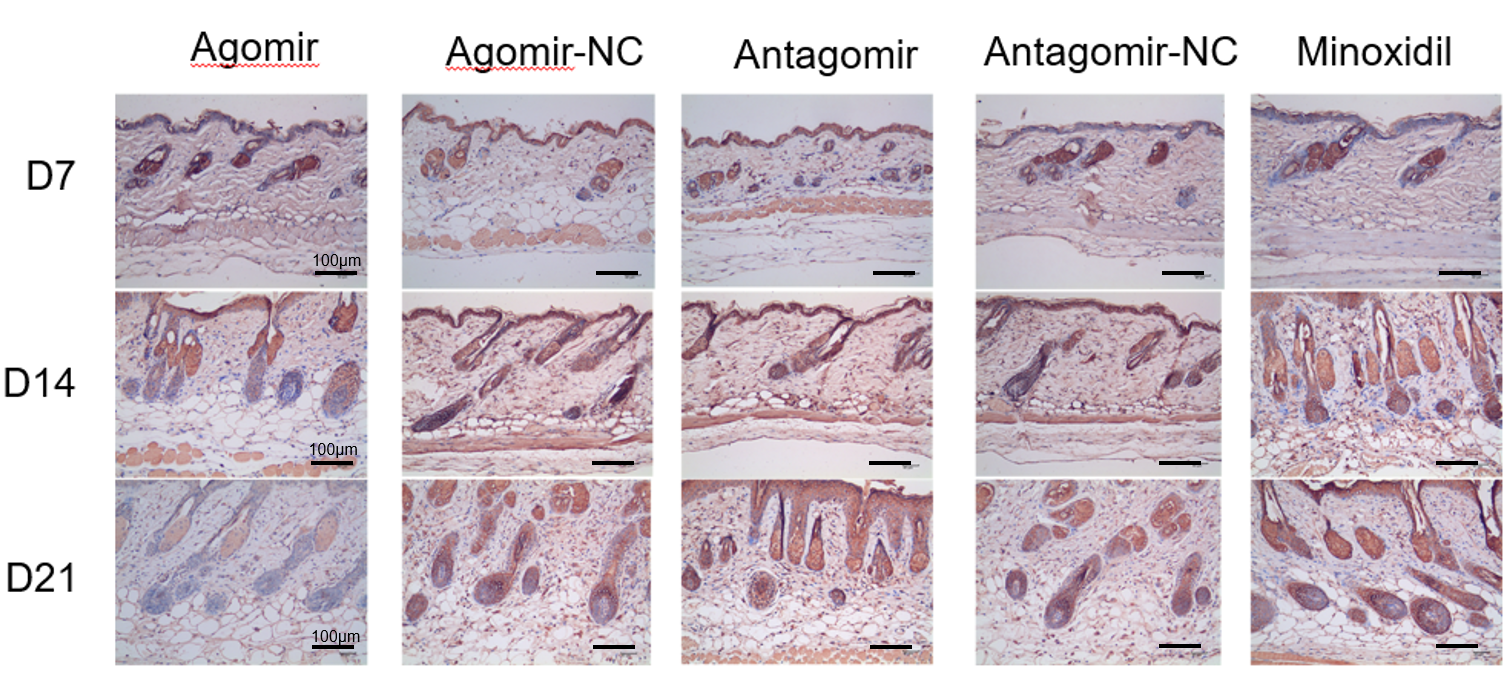


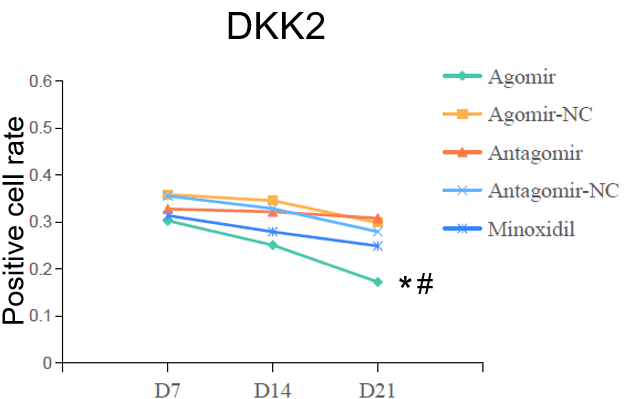


**Figure S24.** Immunohistochemical images and statistical results of DKK2.

Each group showed positive expression on the hair follicles; over time, there were varying degrees of reduction in each treatment group, with the Agomir group showing the most significant reduction, and its expression level was significantly lower than that of the other groups. (scale = 100 μm; n = 3; mean ± SD; one-way ANOVA; * indicates *p* < 0.01 compared with the negative control group: agomir-NC group; # indicates *p* < 0.05 compared with the minoxidil group).


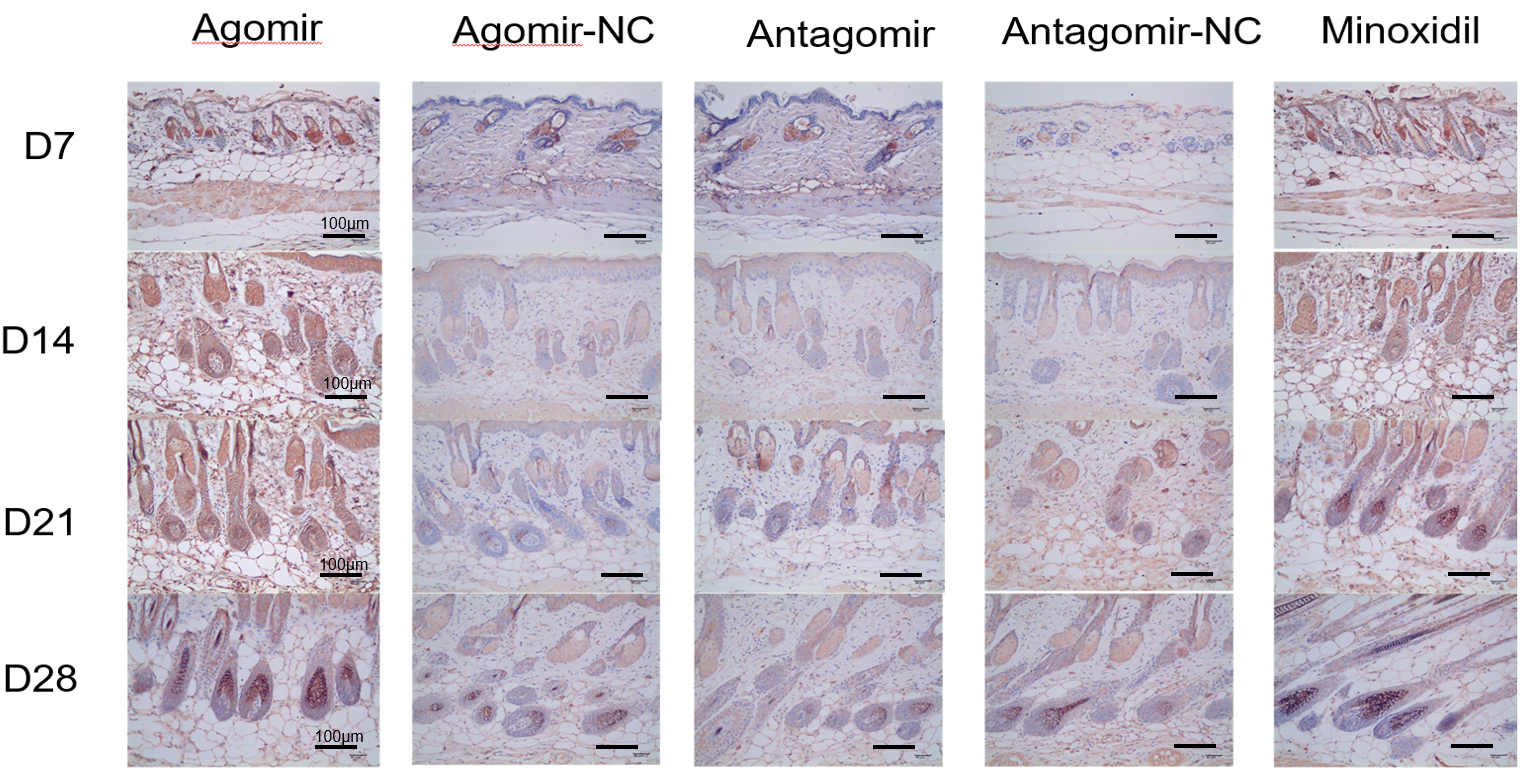


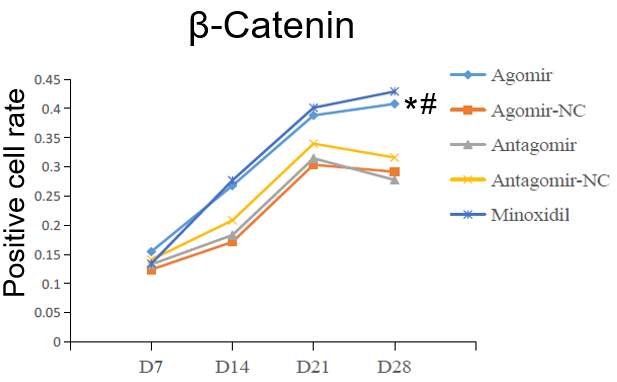


**Figure S25.** Immunohistochemical images and statistical results of β-catenin.

The epidermal and hair follicle outer root sheath cells in different treatment groups were all weakly expressed on the 7th day. With the passage of time, the expression levels of β-catenin in the Agomir group and the minoxidil group increased rapidly, and the rate of increase slowed down by the 21st day. Among them, the positive cell fraction in the minoxidil group was the highest among all groups. (scale = 100 μm; n = 3; mean ± SD; one-way ANOVA; * indicates *p* < 0.01 compared with the compared with the negative control group: agomir-NC; # indicates *p* < 0.05 compared with the minoxidil group).


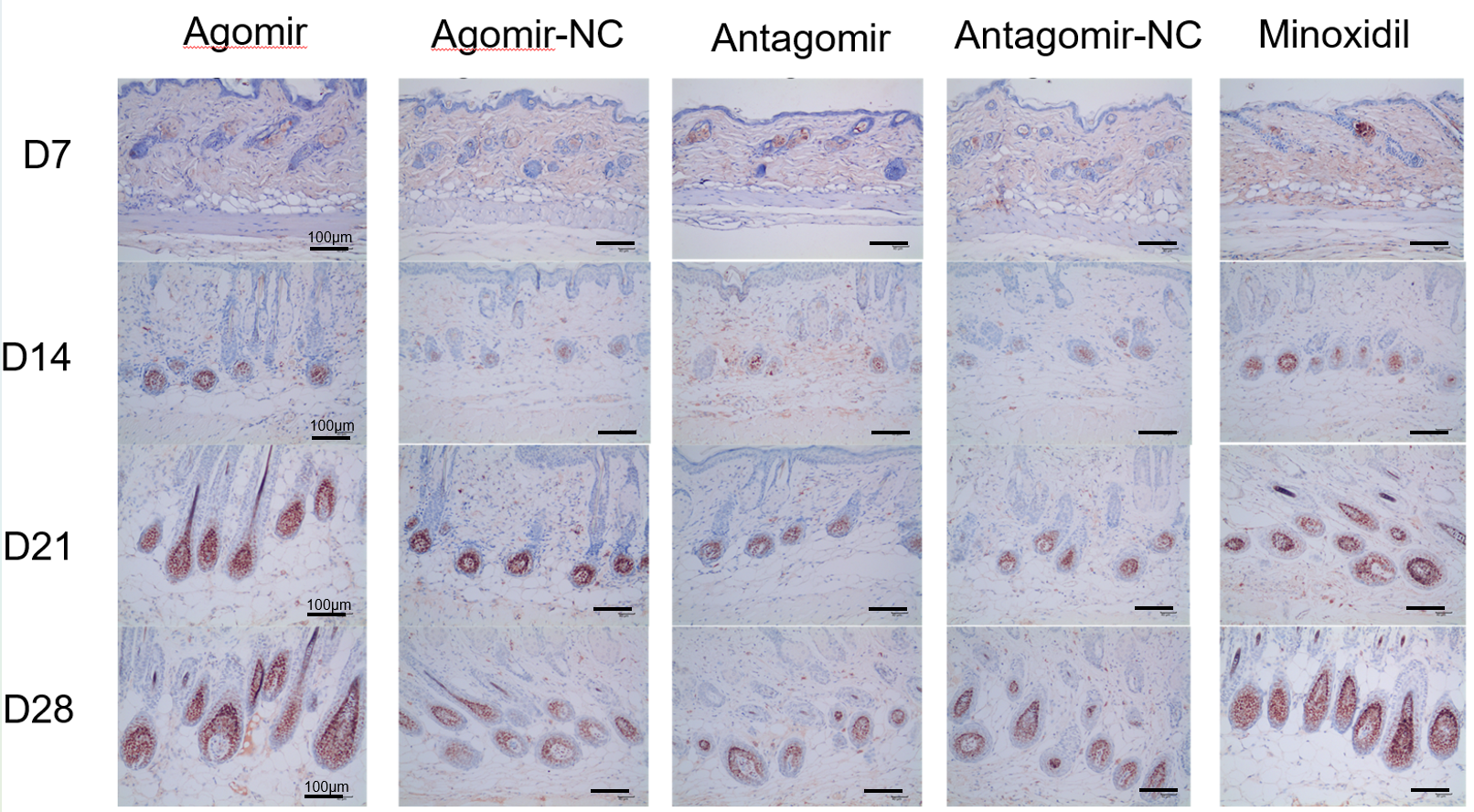


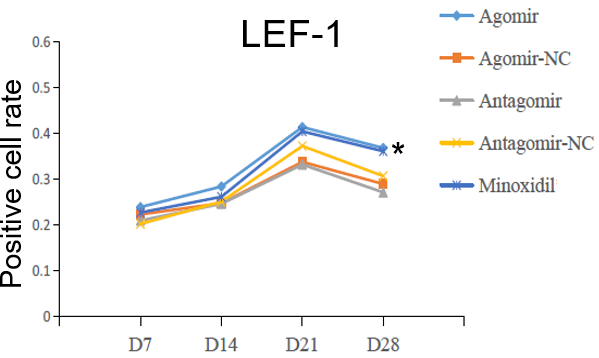


**Figure S26.** Immunohistochemical images and statistical results of LEF1

Starting from the 14th day, expression was observed in the cell nuclei of the hair follicle bulbs in each group. The positive cell fractions in the Agomir group and the minoxidil group increased over time, reaching the highest level on the 21st day, and then decreased on the 28th day. There was no significant difference in expression at the four time points between the two groups, but both were higher than those in the blank and Antagomir groups. (scale = 40 μm; n = 3; mean ± SD; one-way ANOVA; * indicates *p* < 0.01 compared with the negative control group: Agomir-NC).


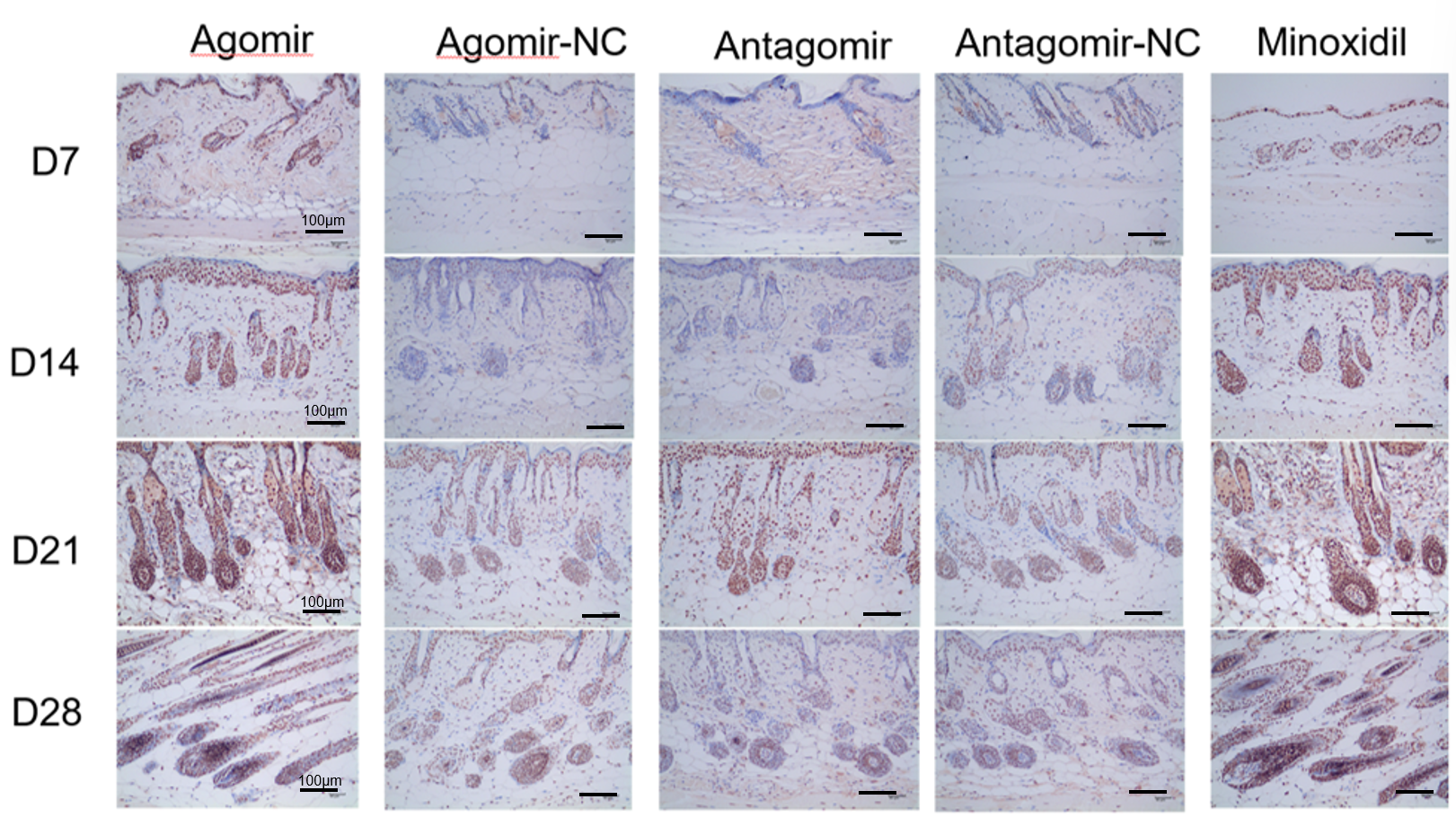


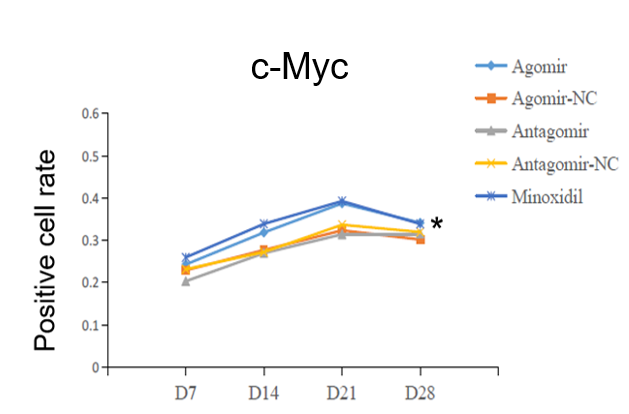


**Figure S27.** Immunohistochemical images and statistical results of C-myc.

The expression of Cmy-c was observed in the nuclei of hair follicle outer root sheath cells and epidermal cells in all treatment groups. During the first 21 days, the expression level of C-myc increased over time, reaching a peak on the 21st day, and then decreased on the 28th day. On the 28th day, there was no statistically significant difference in the proportion of Cmy-c-positive cells between the agomir group and the other groups. (scale = 40 μm; n = 3; mean ± SD; one-way ANOVA; * indicates *p* < 0.05 compared with the negative control group: Agomir-NC).


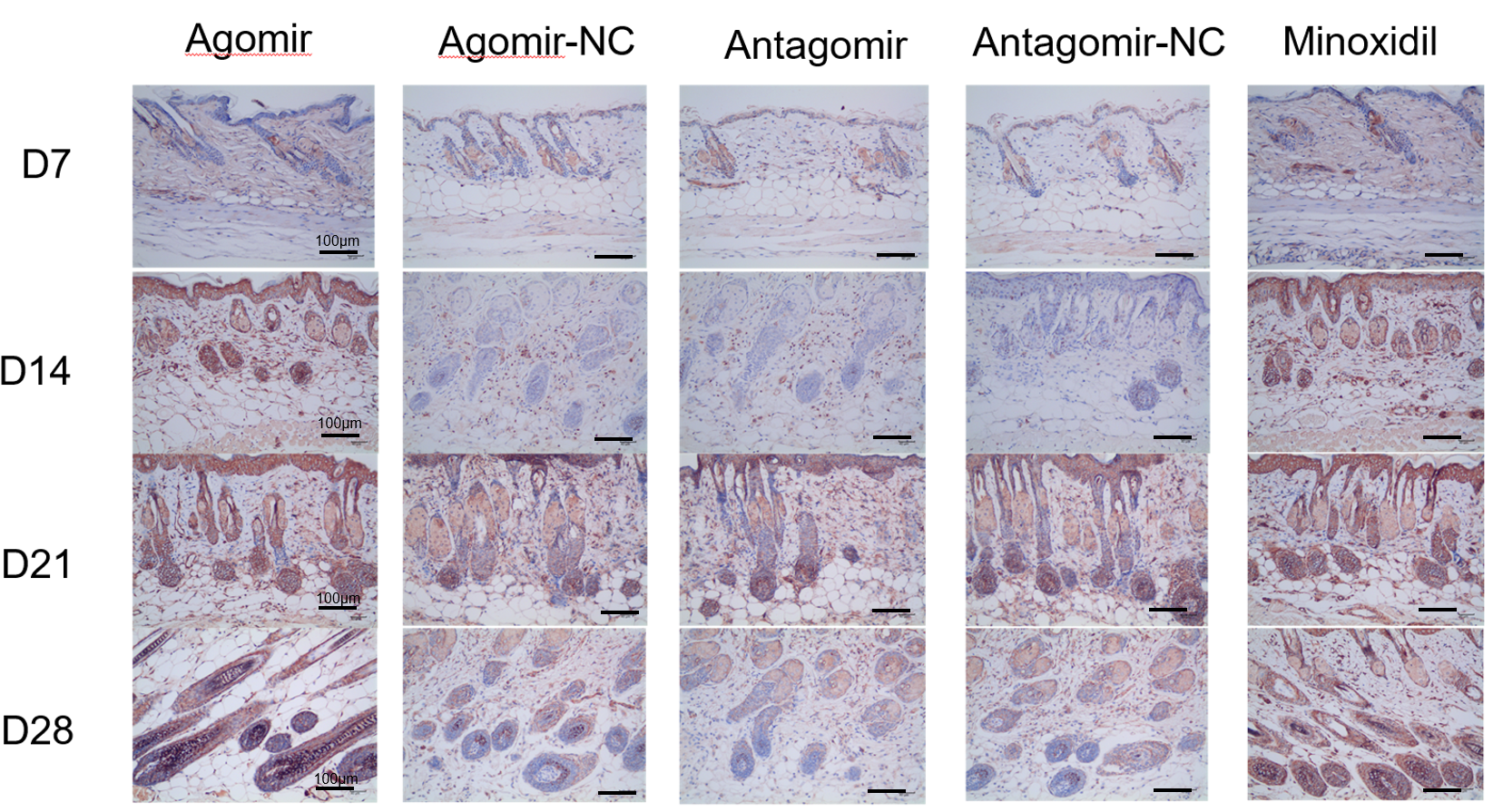


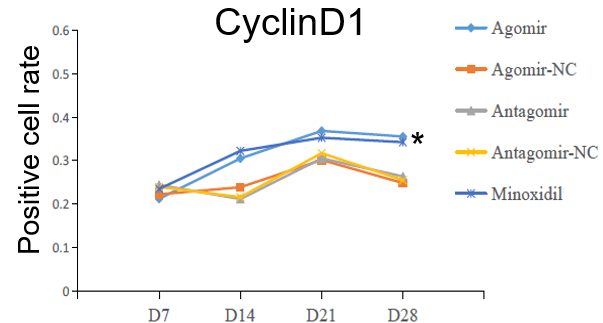


**Figure S28.** Immunohistochemical images and statistical results of CyclinD1.

The expression was observed in the nuclei of hair follicle outer root sheath cells and epidermal cells in all treatment groups. For the first 21 days, the expression level of CyclinD1 increased over time, reaching its peak on the 21st day, and then decreased on the 28th day. The proportion of CyclinD1-positive cells was higher than that in the negative control group and the Antagomir group, and was comparable to that of minoxidil. (scale = 40 μm; n = 3; mean ± SD; one-way ANOVA; * indicates *p* < 0.01 compared with the negative control group: Agomir-NC).


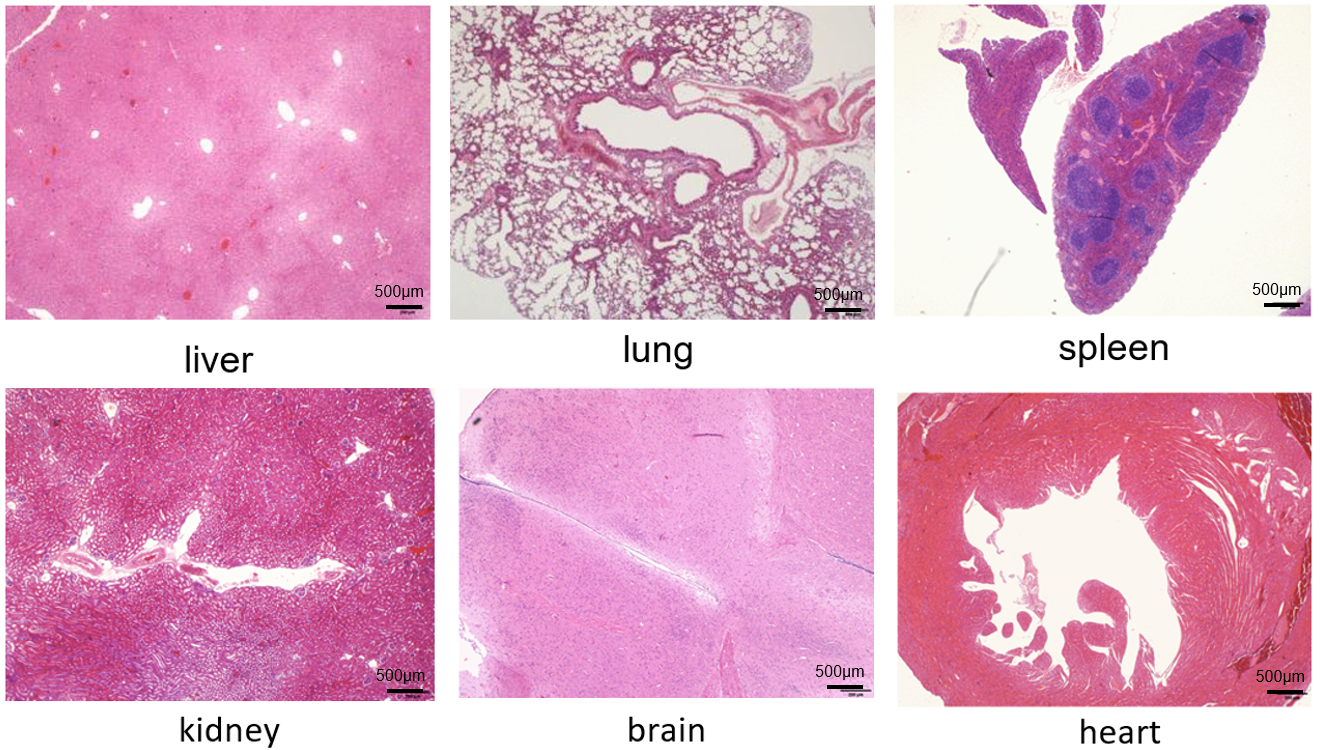


**Figure S29.** Hematoxylin and eosin staining images of multiple organs in agomir group at 28 days.

It can be observed that in the miRNA-221-3p agomir group of mice, after 28 days, tissue sections of each organ were taken for HE staining, and no pathological changes were found in any of the organs. (scale = 500 μm)

1. **eTable section**

| **Primer name** | **Primer sequence（5’-3’）** | **TM（℃）** | **GC %** | **Product length（BP）** |
| --- | --- | --- | --- | --- |
| LEF1-F | TGGTCCCCACACAACTG | 64.1 | 58 | 113 |
| LEF1-R | TCTGTTCATGCTGAGGCTT | 64.1 | 47 |  |
| TCF3-F | TGTCCTTCACTCCTTTGCC | 64.7 | 52 | 128 |
| TCF3-R | GCCCTCTGGTGTAATGGG | 64.7 | 61 |  |
| WNT10B-F | GCGAATCCACAACAACAGGG | 59 | 55 | 291 |
| WNT10B-R | ATAGTGGGGTCTCGCTCACA | 60 | 55 |  |
| CTNNB1-F | CTGAGGAGCAGCTTCAGTCC | 60 | 60 | 161 |
| CTNNB1-R | CCATCAAATCAGCTTGAGTAGCC | 60 | 47 |  |
| GAPDH-F | ACCACAGTCCATGCCATCAC | 60 | 60 | 44 |
| GAPDH-R | TCCACCACCTGTTGCTGTA | 60 | 47 |  |

**Table S1:** Detailed information of primer sequence.

|  |  | **htSKP-EVs** | | | | **hFB-EVs** | | | |
| --- | --- | --- | --- | --- | --- | --- | --- | --- | --- |
| **Type** | **Class** | **Total** | **% of**  **Total** | **Uniq** | **% of**  **Uniq** | **Total** | **% of**  **Total** | **Uniq** | **% of**  **Uniq** |
| **Raw reads** | **NA** | **11359377** | **100.00** | **1253896** | **100.00** | **15500887** | **100.00** | **1197339** | **100.00** |
| **3ADT&length filter** | **Sequence type** | **6749992** | **59.42** | **826554** | **65.92** | **11866193** | **76.55** | **1031102** | **86.12** |
| **Junk reads** | **Sequence type** | **55355** | **0.49** | **5168** | **0.41** | **95033** | **0.61** | **4470** | **0.37** |
| **Rfam** | **RNA class** | **1135002** | **9.99** | **15286** | **1.22** | **602809** | **3.89** | **7316** | **0.61** |
| **mRNA** | **RNA class** | **444442** | **3.91** | **7285** | **0.58** | **511878** | **3.30** | **4498** | **0.38** |
| **Repeats** | **RNA class** | **40651** | **0.36** | **644** | **0.05** | **32454** | **0.21** | **611** | **0.05** |
| **valid reads** | **Sequence type** | **3193144** | **28.11** | **402493** | **32.10** | **2571804** | **16.59** | **151671** | **12.67** |
| **rRNA** | **RNA class** | **552124** | **4.86** | **8941** | **0.71** | **198193** | **1.28** | **3864** | **0.32** |
| **tRNA** | **RNA class** | **493683** | **4.35** | **4635** | **0.37** | **324479** | **2.09** | **2603** | **0.22** |
| **SnoRNA** | **RNA class** | **2573** | **0.02** | **71** | **0.01** | **1210** | **0.01** | **29** | **0.00** |
| **SnRNA** | **RNA class** | **3810** | **0.03** | **160** | **0.01** | **3279** | **0.02** | **44** | **0.00** |
| **other Rfam** | **RNA class** | **82812** | **0.73** | **1479** | **0.12** | **75648** | **0.49** | **776** | **0.06** |

**Table S2: Small-RNA sequencing quality control and read annotation summary for htSKP-EVs and hFB-EVs.**

**References**

1. Acute lymphoblastic leukemia-derived extracellular vesicles affect quiescence of hematopoietic stem and progenitor cells. Cell Death Dis. 2022 Apr 12;13(4):337.
2. Small extracellular vesicles ameliorate peripheral neuropathy and enhance chemotherapy of oxaliplatin on ovarian cancer. J Extracell Vesicles.2021 Mar;10(5):e12073.
3. Vitamin A-coupled stem cell-derived extracellular vesicles regulate the fibrotic cascade by targeting activated hepatic stellate cells invivo. J Control Release. 2021 Aug 10;336:285-295.
